# Transplantation of hiPSC-derived pericytes rescues Alzheimer’s disease phenotypes in *APOE*4/4 mice through IGF2-rich apoptotic vesicles

**DOI:** 10.1186/s40035-025-00512-6

**Published:** 2025-11-13

**Authors:** Chuanfeng Xiong, Yao Tang, Junhua Chen, Mingming Fan, Lan Wei, Zhaoran Dong, Xingqiang Lai, Xuejiao Men, Qiumin Chen, Dairui Li, Wenjin Ye, Yuanchen Ma, Xiaoyong Chen, Weijun Huang, Zhengqi Lu, Hong Chen, Yunfeng Shen, Yanming Chen, Andy Peng Xiang, Weiqiang Li

**Affiliations:** 1 Department of Endocrinology and Metabolic Diseases, The Eighth Affiliated Hospital of Sun Yat-Sen University, Shenzhen, 518033 Guangdong China; 2 Center for Stem Cell Biology and Tissue Engineering, Key Laboratory for Stem Cells and Tissue Engineering, Ministry of Education, Sun Yat-Sen University, Guangzhou, 510080 China; 3 Present Address: Center for Stem Cells and Regenerative Medicine, National-Local Joint Engineering Research, Zhongshan School of Medicine, Sun Yat-Sen University, Guangzhou, 510080 China; 4 Department of Histoembryology and Cell Biology, Zhongshan School of Medicine, Sun Yat-Sen University, Guangzhou, 510080 China; 5 Department of Neurology, The Third Affiliated Hospital, Sun Yat-Sen University, Guangzhou, 510630 China; 6 Center for Stem Cells Translational Medicine, Shenzhen Qianhai Shekou Free Trade Zone Hospital, Shenzhen, 518067 China; 7 Department of Endocrinology and Metabolic Diseases, The Third Affiliated Hospital of Sun Yat-Sen University, Guangzhou, 510630 China; 8 Guangdong Provincial Key Laboratory of Diabetology & Guangzhou Municipal Key Laboratory of Mechanistic and Translational Obesity Research, The Third Affiliated Hospital of Sun Yat-Sen University, Guangzhou, 510630 China

**Keywords:** Alzheimer’s disease, *APOE*4, Pericytes, ApoVs, IGF2

## Abstract

**Background:**

Effective therapies for Alzheimer’s disease (AD) remain to be developed. *APOE4* is the strongest genetic risk factor for late-onset AD. Pericyte degeneration and blood–brain barrier (BBB) disruption are thought to be early biomarkers of AD and contribute to cognitive decline in *APOE4* carriers, representing potential therapeutic targets. Our previous studies have shown that pericyte transplantation is one of the most effective strategies for BBB restoration, exhibiting great therapeutic potential for *APOE4*-related BBB damage and AD phenotypes.

**Methods:**

*APOE*4/4 mice were treated with pericytes derived from *APOE*3/3 human induced pluripotent stem cells (hiPSCs). Behavioral tests, AD pathologies, and BBB integrity were assessed. Subsequently, temporal and spatial distribution of the transplanted pericytes was analyzed using tdTomato^+^ lentivirus labeling. Next, therapeutic effects of apoptotic vesicles (ApoVs) generated from *APOE*3/3 pericytes were evaluated in *APOE*4/4 pericytes in vitro. Additionally, transcriptomic and proteomic profiling were performed to identify key effector molecules in pericyte-derived ApoVs. Finally, the therapeutic effects of ApoVs derived from pericytes were evaluated in *APOE*4/4 mice.

**Results:**

Early, multiple transplantations of pericytes derived from *APOE*3/3 hiPSCs robustly rescued cognitive decline and AD pathologies, restored BBB integrity, and prevented in situ pericyte degeneration in aged *APOE*4/4 mice. Intriguingly, ApoVs released from the infused cells, rather than the transplanted pericytes, were predominantly distributed in the brain, which were ingested by in situ* APOE*4/4 pericytes and then promoted functional recovery. We further characterized insulin growth factor-2 (IGF-2) as a key factor in *APOE*3/3 pericyte-derived ApoVs. Infusion of the in vitro generated ApoVs from *APOE*3/3 pericytes demonstrated distinct therapeutic effects in *APOE*4/4 mice, which were reversed by IGF2 knockout.

**Conclusions:**

*APOE*3/3 pericytes or *APOE*3/3 pericyte-derived IGF2-rich ApoVs may offer promising therapeutic strategies for *APOE*4-associated AD.

**Supplementary Information:**

The online version contains supplementary material available at 10.1186/s40035-025-00512-6.

## Background

Alzheimer’s disease (AD), characterized by neuronal loss and cognitive decline, is the most common type of dementia [1]. Globally, over 50 million individuals suffer from AD, and the number doubles every 20 years [2]. Since description of the first case in 1906, abnormal extracellular deposition of amyloid-β (Aβ) and intracellular accumulation of hyperphosphorylated tau (p-tau) in neuronal cells remain the leading theory of AD-related pathologies [3]. Thus, therapeutic strategies targeting Αβ have been considered as the prime choice for AD drug development. Two monoclonal antibodies for Αβ, lecanemab and donanemab, have been approved by US Food and Drug Administration for clearing Αβ plaques and decelerating cognitive decline of AD patients in the early stages [4]. However, the clinical benefits of Αβ-targeting treatments remain modest, with other limitations of high cost and side effects. Therefore, it is still urgent to unravel the pathophysiological mechanisms underlying AD and develop new therapeutic choices for the treatment of AD.

While the specific causes of AD are not fully understood, the apolipoprotein E allele 4 (*APOE4*) has been recognized as the strongest genetic risk factor for late-onset AD, accounting for almost 50% of AD patients in the United States [5]. People carrying two copies of the *APOE* ε4 allele have a higher risk of AD and earlier disease onset than heterozygous individuals. The *APOE* gene product, ApoE, is a plasma lipoprotein which plays a key role in lipid circulation and metabolism [6]. There are three ApoE isoforms: ApoE2 (Cys112/Cys158), ApoE3 (Cys112/Arg158), and ApoE4 (Arg112/Arg158), which differ only by a single amino acid change between Cys and Arg [7]. Compared with the most common *APOE* ε3 allele, *APOE* ε4 increases, while *APOE* ε2 decreases the risk of AD [8]. From age 55, almost all *APOE*4 homozygotes have higher levels of AD biomarkers compared to *APOE*3 homozygotes [9]. Nevertheless, effective therapies for *APOE*4-related AD have not yet been reported. Even lecanemab is carefully recommended for *APOE*4-carriers due to the higher risk of amyloid-related imaging abnormalities with edema or effusions [10].

The blood–brain barrier (BBB) separates circulating blood from neural tissues through a selective semi-permeable membrane, preventing neurotoxic plasma components, blood cells, pathogens and other neurotoxic substances from the brain [11]. Nevertheless, studies indicate that *APOE4* accelerates BBB breakdown and causes degeneration of brain capillary pericytes, contributing to cognitive decline independently of Aβ and phosphorylated tau (p-tau) in the cerebrospinal fluid [12, 13]. In *APOE4* mice, increased BBB permeability was also observed as early as 8–12 weeks of age, prior to occurrence of neuronal changes [14]. Brain pericytes, originating from cranial neural crest (CNC) cells during embryonic development, are critical in the establishment and maintenance of the BBB integrity [15]. Experimental ablation of pericytes leads to BBB breakdown, blood-flow reduction, white matter dysfunction and neuronal loss, while implantation of pericytes differentiated from C3H/10T1/2 mouse embryo fibroblasts in the brain cortex enhances cerebral blood flow and reduces Aβ pathology in *APP/PS1* mouse model of AD [16, 17]. Thus, pericyte transplantation may be a promising therapeutic strategy for *APOE4*-related AD.

In our previous research, we successfully derived pericyte-like cells (PCs) from human induced pluripotent stem cells (hiPSCs) through the CNC stage, and demonstrated that intravenous injection of hiPSCs-CNC PCs could efficiently restore BBB integrity in the transient middle cerebral artery occlusion (tMCAO) mouse model [18]. However, whether hiPSCs-CNC PCs could improve BBB barrier function and cognition of *APOE4* carriers remains to be explored. In the current study, we aimed to investigate the effects of transplantation of *APOE*3/3-PCs on behavioral performance, AD-related pathological features, and BBB function in *APOE*4/4 mice. Additionally, the spatial and temporal distribution characteristics of transplanted cells and the underlying mechanisms were investigated.

## Materials and methods

### Animals

Female C57BL/6 (wild-type, WT) mice aged 8–12 weeks (weight 18–25 g) were purchased from GemPharmatech Co., Ltd (Nanjing, China). Homozygous *APOE*4/4 mice were from Cyagen Biosciences Inc. (Suzhou, China) and bred under standard specific-pathogen-free (SPF) conditions. All mice were housed in a temperature-, humidity- and light cycle-controlled facility (20 ± 2 °C; 50% ± 10%; 12-h light/dark cycle) with free access to food and water. For the single injection project, aged (18 months old) *APOE*4/4 mice were intravenously injected with 1 × 10^6^ *APOE*3/3-PCs, human dermal fibroblasts (HDFs; Cat. #2320, ScienCell Research Laboratories, Carlsbad, CA) or an equal volume of phosphate buffered saline (PBS). For the multiple injection project, mice of 2–3 months old were intravenously injected with 1 × 10^6^ *APOE*3/3-PCs, HDFs or an equal volume of PBS once a month for 8 consecutive months. For apoptotic vesicle (ApoV) treatment, *APOE*4/4 mice aged 2–3 months were intravenously injected with 2 × 10^7^ ApoVs^−PCs^, ApoVs^−HDFs^ or an equal volume of PBS every 21 days for 10 times. All animal experimental procedures were approved by the Sun Yat-Sen University Animal Use and Care Committee (2020-000305).

### Generation of hiPSCs

Peripheral blood mononuclear cells (PBMCs) from *APOE*3/3 or *APOE*4/4 carriers were obtained at the Third Affiliated Hospital of Sun Yat-Sen University following the standards of Ethics Committee of the hospital (Approval number: 2020-02-148-01) and the 1964 Helsinki Declaration and its later amendments or comparable ethical standards. *APOE*3/3 and *APOE*4/4 hiPSCs were generated by transduction of human PBMCs with Sendai viral vectors (Cat. #A16517, Thermo Fisher Scientific, Rutherford, NJ) according to the manufacturer’s instructions [19]. The hiPSCs were maintained at feeder-free conditions using mTeSR™ Plus medium (Cat. #100–0274, Stem Cell Technologies, Vancouver, Canada) on pre-coated Matrigel (Cat. #354277, BD Bioscience, San Diego, CA). Cells were passaged every 4–5 days with 0.5 mmol/L EDTA (Cat. # 15575020, Invitrogen, Carlsbad, CA). *APOE* genotyping was performed using a PCR-restriction fragment length polymorphism approach [20]. The *APOE*3/3 and *APOE*4/4 genotypes were confirmed by Sanger sequencing. Information of *APOE*3/3 and *APOE*4/4 carriers is listed in Table S1.

### Pericyte-like cell differentiation from hiPSCs

Pericyte-like cells were differentiated as described in our previous study [18]. In brief, hiPSCs were harvested using Accutase (Cat. # 00-4555-56, Invitrogen) and plated onto a Matrigel-coated T75 cell culture flask at a density of 10^4^ cells/cm^2^. A chemically defined N2B27 medium (N2B27-CDM) containing 95% DMEM/F12 medium, 1% N2 supplement, 2% B27 supplement, 1% Glutamax, 1% MEM nonessential amino acids, 55 μmol/L 2-mercaptoethanol (all from Invitrogen), 10 μmol/L Y27632 (Cat. #72304, Stem Cell Technologies), and 20 ng/ml basic fibroblast growth factor (bFGF) (Cat. #100-18C, Peprotech, Rocky Hill, NJ) was used. Twenty-four hours later, the N2B27-CDM medium was replaced with NCN2 medium containing N2 supplement, CHIR99021 (1 μmol/L), and SB431542 (2.0 μmol/L) and cultured for 6 days. Then the cells were dissociated to single cells using Accutase and labeled with antibodies for CD57 (Cat. #558619, BD Bioscience) and low-affinity nerve growth factor receptor p75 (Cat. #560326, BD Bioscience) for fluorescence-activated cell sorting (FACS). p75^bright^HNK1^+^ CNC stem cells were sorted and replated onto poly-L-ornithine (PO; 15 mg/ml, Cat. #P4957, Sigma-Aldrich, St. Louis, MO)- and fibronectin (FN; 10 mg/ml, Cat. #FC010, Millipore, Temecula, CA)-coated dishes for adherent culture with neural crest culture medium (NCCM) containing 1% N2 supplement, 2% B27 supplement, 20 ng/ml bFGF, and 20 ng/ml epidermal growth factor (EGF; Cat. #AF-100-15, Peprotech). CNCs were dissociated using Accutase and plated onto the PO/FN-coated dishes at a density of 10^5^ cells/cm^2^ in NCCM supplemented with 10 μmol/L Y27632. When differentiation was initiated, the culture was switched to commercial Pericyte Medium (Cat. #1201, ScienCell Research Laboratories) containing 10 ng/ml bFGF and 50 ng/ml platelet derived growth factor BB (Cat. #100-14B, Peprotech) for 7–14 days. The first confluent culture at this time point was denoted as passage 1. Cells were dissociated by Accutase solution, split at 1:3 in Pericyte Medium, and cultured on PO/FN-coated plates.

## Behavior tests

### Open field test (OFT)

Animals were moved to the testing room at least 3 h prior to the test. OFT was performed in a cubic open field chamber (50 cm × 50 cm × 50 cm), with bottom area divided into a central field (25 cm × 25 cm) and a peripheral field. Mouse behaviors were captured using a SONY HDRCX405 video camera for 10 min and analyzed by a behavioral analysis software (TopScanLite, Clever System, Reston, VA). The total distance, center distance, number of entries in the center and the time spent in the central field were recorded for analysis.

### Morris water maze (MWM)

MWM was conducted in a circular tank (diameter, 120 cm) filled with opaque water at a temperature of 24 °C. Four visual cues with different shapes were placed around the tank to assist recognition of orientation. The water was made opaque with a non-toxic chemical titanium dioxide (food-grade titanium dioxide). The platform was submerged ~ 1.0 cm below the water surface. During the training phase, mice were trained once a day for 5 consecutive days. During training, each mouse was given 60 s to find the platform. If it failed to find the platform within 60 s, it would be guided to the platform and stayed there for 15 s. In the spatial probing test on day 6, the platform was removed and each mouse was allowed to swim for 60 s. The time and the number of crossings in the platform quadrant were recorded. Mouse behavior was recorded by a SONY HDRCX405 video camera and analyzed using the TopScanLite software (TopScanLite, Clever System, Reston, VA).

### New object recognition (NOR)

NOR was conducted in a cubic open field chamber (50 cm × 50 cm × 50 cm). Briefly, mice were placed in the chamber and allowed for free exploration for 10 min on day 1 (habituation period). Twenty-four hours later, two familiar objects were placed in the chamber and mice were allowed to explore for 10 min (learning period). After another 24-h interval, one familiar (F) object was replaced by a novel object (N), and mice were allowed to explore the two objects for 10 min (testing period). The time spent exploring the familiar and novel objects and animal movement were recorded using the TopScanLite software. Recognition index of exploration was calculated as follows: recognition index = time spent on the new object/total exploration time of the two objects.

### Spontaneous alternation T-maze

Spontaneous alternation T-maze test was used to assess the spatial working memory of mice. The T-maze was a T-shaped apparatus featuring a start arm and two goal arms. Each arm was endowed with a guillotine door. Each mouse was transferred to the testing room at least 3 h prior to the test. During each trial, the animal was first placed in the start arm for 30 s. Then the guillotine door of the start arm was opened. When the mouse entered a goal arm, the guillotine door behind it was closed. Then, the mouse was placed back to the start arm again for 30 s, and then the guillotine door of the start arm was opened to allow the mouse to make a choice between the two goal arms. Trials were marked as successful if the mouse chose different goal arms on each run. Alternation rate was defined as the total proportion of successful trials for each animal during 10 consecutive trials.

### Establishment of tdTomato^+^ PCs and mCherry^+^EGFP^+^ PCs

Early passage of PCs was transduced with hU6-MCS-Ubiquitin-tdTomato-IRES-puromycin control lentivirus (GeneChem, Shanghai, China). Forty-eight hours later, puromycin was used to select stable tdTomato^+^ cells for cell tracing in vivo. mCherry^+^EGFP^+^ lentivirus was kindly provided by Xiaoran Zhang’s lab (Zhongshan School of Medicine, Sun Yat-sen University) synthesized according to the methods published previously [21]. Briefly, the sLP-mCherry sequence and the EGFP sequence were cloned into a pRRL lentiviral backbone. A soluble peptide and a modified TAT peptide were cloned upstream of the mCherry cDNA (sLP-mCherry), in which sLP–mCherry is used to label phospholipid bilayer and EGFP represents cell contents. PCs were stably infected with lentiviral particles. mCherry^+^EGFP^+^ PCs were used for tracing PC-released EVs in vivo.

### Multiphoton microscopic analysis

Multiphoton microscopy was performed to detect BBB leakage in vivo. In brief, mice were anesthetized with isoflurane and a cranial window (~ 0.5 cm in diameter) was made. Fluorescein isothiocyanate (FITC)-conjugated dextran (Cat. #46944, Sigma-Aldrich) was injected via tail vein (1.5 mg per mouse). In vivo time-lapse images were acquired for a total of 30 min using an FVMPE-RS confocal microscope (Olympus, Tokyo, Japan). The fluorescence intensity of dextran was quantified by Image J software (https://imagej.nih.gov/ij/). To observe ApoV release from the lung of *APOE*4/4 mice, fresh dissected lung was placed in a 6-well plate and perfused with PBS. ApoVs released from lung were observed under the FVMPE-RS confocal microscope, and time-lapse images were acquired every 5 min for 1 h.

### Isolation and characterization of ApoVs

ApoVs were induced as previously reported [22]. When PCs or HDFs reached full confluency, the culture medium was removed and replaced with a basal medium containing 500 nmol/L staurosporine (STS) (Cat. #SB17469, Macklin, Shanghai, China) without FBS. After induction of apoptosis for 12 h, ApoV-containing supernatant was isolated and ApoVs were purified using sequential centrifugation. Briefly, cells and cell debris were removed by 800 × *g* centrifugation for 10 min and 2000 × *g* centrifugation for another 10 min. Next, the supernatant was centrifuged at 15,000 × *g* for 30 min to pellet the ApoVs. Cleaved-caspase-3 assay and flow cytometry analysis with annexin V and PI staining were used to evaluate the apoptotic rate of cells. The morphology of ApoVs was observed with transmission electron microscopy (TEM) (HT7800, Hitachi High-Technologies Corporation, Tokyo, Japan). ApoV surface markers CD9, CD63, and CD81 were assessed by flow cytometry.

### Dextran leakage and Aβ_1-40_ transcytosis assay

To test the barrier function of PCs and the treatment effect of ApoVs, PCs were cocultured with primary human brain microvascular endothelial cells (HBMECs) in 24-well transwell inserts (Cat. #3413, Corning Life Sciences, Topsfield, MA) pre-coated with collagen (1 μg/ml) and FN (10 μg/ml) for at least 4 h at 37 °C. PCs (10,000 cells per insert) and HBMECs (20,000 cells per insert) were seeded on the top side of the inserts and cocultured for 48 h. For ApoVs treatment, 2 × 10^5^ ApoVs derived from *APOE3/3*-PCs or HDFs were added to the upper chamber of the transwell.

To assess permeability, 4-kD dextran labeled with FITC was added to the upper chamber of the transwell and placed on a shaker in an incubator. Two hours later, medium in the bottom well was collected for analysis. For Aβ_1-40_ transcytosis assay, FITC-labeled Aβ_1-40_ was added into the lower chamber. The medium in the upper compartment was collected after 24 h. Fluorescent intensity was then analyzed with a fluorometric plate reader (Infinite F200 Pro, Tecan Life Sciences, Männedorf, Switzerland) at 488 nm excitation and 525 ± 20 nm emission.

### Immunohistochemistry (IHC) and immunofluorescence (IF) analysis

For IF analysis, mouse brain samples were fixed in 4% paraformaldehyde (PFA) overnight at 4 °C, washed with PBS and then immersed in 30% sucrose solution overnight at 4 °C. Samples were embedded in optimal cutting temperature compound (OCT, Cat. #4583, Sakura Finetek, Torrance, CA) and cut into 35 μm-thick sections. The sections were blocked in blocking buffer (5% donkey serum in 0.3% Triton X-100 in PBS) for 1 h at room temperature, incubated with primary antibodies overnight at 4 °C and then secondary antibodies for 2–4 h at room temperature. For cell tracing experiments, brain samples were sliced at 50 μm thickness to better trace and identify the complete cellular structures. For in vitro samples, cells were fixed with 4% PFA at room temperature for 20 min and washed three times with PBS. Then, cells were permeabilized with 0.3% Triton X-100 in PBS and incubated overnight at 4 °C with primary antibodies and with secondary antibodies for 1–2 h at room temperature.

The following antibodies were used: NeuN (Abcam, Cat. #ab177487, 1:200, Cambridge, UK), Iba1 (Genetex, Cat. #GTX100042, 1:100, Irvine, CA), Fibrinogen (Abcam, Cat. #ab43269, 1:100), Albumin (Abcam, Cat. #ab207327, 1:100), ZO1 (Thermo Fisher Scientific, Cat. #40-2200, 1:100, Waltham, MA), Occludin (Thermo Fisher Scientific, Cat. #71-1500, 1:100, Waltham, MA), Lectin (Vector, Cat. #DL-1178-1, 1:100, San Ramon, CA), Pfgfrβ (Cell Signaling Technology, Cat. #3169, 1:100, Danvers, MA), cleaved-caspase3 (Abcam, Cat. #ab2302, 1:100), and CD206 (Biolegend, Cat. #141708, 1:50, San Diego, CA). DAPI (Roche, Cat. #1023627001, Basel, Switzerland) was used as a counterstain for the nucleus.

Images of NeuN/Iba1 staining, fibrinogen/albumin leakage, pericyte coverage and ZO1/Occludin coverage were reconstructed as maximum projections of 10-μm-thick Z-stack images using Imaris Viewer (Oxford, Oxford, UK), and subsequent analyses were performed using ImageJ software (NIH, Bethesda, MA). For each animal, 5 sections corresponding to the plane of maximum coronal surface area were collected. For quantification, five randomly selected views of either the cortex or hippocampus were captured from each section, and the statistical values were derived based on the average of 25 views (5 sections × 5 images per section). The experiments were repeated three times to ensure reproducibility.

For IHC analysis, mouse brain samples were embedded in paraffin and cut into 5 µm-thick coronal sections. Tissue sections were incubated overnight with primary antibodies: anti-Aβ40 Rabbit pAb (Servicebio, Cat. #GB111197-100, 1:100, Wuhan, China), and phospho-Tau-T181 Rabbit mAb (ABclonal, Cat. #AP1387, 1:100). Staining was revealed using biotinylated secondary antibodies (Servicebio, Cat. #GB1302) and the ABC-HRP kit (Cat. #PK-4000, Vector, San Ramon, CA). Aβ burden (refers to the proportion of Aβ-positive area to the captured area) and the number of p-tau-positive cells per unit area (mm^2^) were quantified. For each animal, five coronal sections were collected. For each section, five images were randomly acquired from either the cortex or the hippocampus. Thus, for each animal, a total of 25 images (5 sections × 5 images per section) were analyzed, and statistical values were calculated based on the average across these 25 images.

### Flow cytometry

Flow cytometry was performed using the CytoFLEX flow cytometer, and results were analyzed with the CytExpert (Beckman, Brea, CA). The following antibodies were used: FITC anti-human CD9 antibody (1:100; Cat. #312103, Biolegend), PE/Cyanine7 anti-human CD63 antibody (1:100; Cat. #353009, Biolegend), APC anti-human CD81 antibody (1:100; Cat. #349509, Biolegend), and BD Pharmingen™ FITC Annexin V Apoptosis Detection Kit I (Cat. #556547, BD Biosciences).

### RNA sequencing (RNA-seq)

RNA-seq was performed as previously reported [18]. Total mRNA was isolated from ApoVs^−HDFs^ or ApoVs^−PCs^ for bulk RNA-seq analysis. RNA-seq libraries were constructed using the Illumina mRNA-seq Prep Kit (Cat. #20020594, Illumina, San Diego, CA) according to the instructions of the manufacturer. The fragmented and randomly primed 150 bp paired-end libraries were sequenced using Illumina Novaseq 6000 (Illumina). Sequencing data were processed using Consensus Assessment of Sequence and Variation (CASAVA, version 1.8.2; Illumina) using the default settings. The TPM values were used to evaluate the expression levels of genes. Pearson’s correlation coefficients were calculated (*R*^2^) to measure the similarities of the global gene expression profiles between ApoVs^−HDFs^ and ApoVs^−PCs^. Finally, the RNA-Seq data were analyzed using the Ingenuity Pathways Analysis software (Ingenuity Systems Inc., Redwood City, CA) to categorize the differentially regulated genes. RNA-seq data have been deposited at the NGDC database (https://ngdc.cncb.ac.cn/gsa-human/) under accession number HRA009346.

### Proteomic analysis

4D label-free quantitative proteomics analysis was supported by Jingjie PTM BioLabs (Hangzhou, China), including protein extraction, trypsin digestion, HPLC fractionation and LC-MS/MS analysis. Proteins were identified by comparing against the Uniprot database with a false discovery rate (FDR) set at 0.01 for both peptides and proteins. Proteins with differential expression between ApoVs^−HDFs^ and ApoVs^−PCs^ were included for further functional analysis. The details of all the identified proteins are listed in Additional file 2, Table S2.

### IGF2 knockout in *APOE*3/3-PCs

Gene knockout was performed using the CRISPR–Cas9 system. pLenti-IGF2-sgRNA (Cat. #L27870) and pLenti-Control-sgRNA (Cat. #L00011) were purchased from Beyotime Biotechnology (Shanghai, China).

To improve transfection efficiency of the cells, the plasmid together with packaging plasmids (psPAX2 and pMD2.G) were co-transfected into HEK293FT cells for lentivirus packaging at a ratio of 3:2:1 using Lipofectamine 3000 transfection reagent (Cat. #L3000008, Thermo Fisher Scientific, Waltham, MA) according to the manufacturer’s protocol. Briefly, the HEK293FT cells were seeded in complete DMEM medium (Cat. #C11995500BT, Gibco, Grand Island, NY) to reach 70%–80% confluence at the time of transfection. The plasmid DNA was diluted in Opti-MEM medium and mixed with the Lipofectamine 3000 reagent. Subsequently, the DNA mixture was added dropwise to the cell culture medium. The virus-containing supernatant was collected at 48  and 72 h post-transfection, filtered through a 0.22-μm PVDF membrane (Cat. #SLGV033RB, Millipore, Temecula, CA) and enriched by centrifuging at ~ 50,000 × g for 2 h. Finally, early passage of *APOE*3/3-PCs were infected, and the knockout efficiency of each sgRNA-containing lentivirus was assessed by western blot.

### Quantitative PCR analysis

Total RNA was extracted from ApoVs^−HDFs^ and ApoVs^−PCs^ using TRIzol Reagent (Invitrogen) according to the manufacturer’s instructions. RNA concentrations were determined using the NanoDrop ND-1000 spectrophotometer (NanoDrop Technologies, Wilmington, DE). Total RNA (1 μg) was converted to cDNA using a Quantitect Reverse Transcription kit (Qiagen, Valencia, CA). Quantitative real-time PCR (qPCR) analysis was performed using the DyNAmo ColorFlash SYBR Green qPCR kit (Cat. #F416-L, Thermo Fisher Scientific, Waltham, MA) and the LightCycler 480 Detection System (Roche Diagnostics, Branchburg, NJ). The expression levels were normalized to glyceraldehyde-3-phosphate dehydrogenase (GAPDH), and changes in gene expression were calculated as fold changes using the ΔΔCt method. Primer used in this study were: *IGF2* forward CGCTTCAGTTTGTCTGTTCG, reverse GCAGCACTCTTCCACGATG; *GAPDH*: forward GAACATCATCCCTGCATCCA, reverse CCAGTGAGCTTCCCGTTCA.

### Western blotting analysis

For western blot analysis, half mouse brain or cell samples were washed with cold PBS, lysed in 1 × RIPA buffer and then centrifuged at 15,000 × *g* for 10 min at 4 °C. Protein concentration was measured using the Pierce BCA Protein Assay Kit (Cat. #23225, ThermoFisher Scientific, Waltham, MA). Equal amounts of protein were separated by sodium dodecyl sulfate polyacrylamide gel electrophoresis and then electrotransferred to 0.45-μm pore-sized polyvinylidene difluoride membranes (Cat. #HVLP04700, Millipore, Burlington, MA). Each membrane was blocked in 0.1% (*v*/*v*) Tween 20/TBS (TBS/T) containing 5% (*w*/*v*) nonfat milk powder for 1 h at room temperature, incubated with appropriate primary antibodies overnight at 4 °C and then with peroxidase-coupled secondary antibodies. Proteins were detected with ECL substrate Kit (Cat. #JY01054, Jiangyuan Bio, Nanjing, China). All Western blot experiments were performed in triplicate. The primary antibodies were anti-Aβ_40_ (Servicebio, Cat. #GB111197-100, 1:1000), Aβ1-42 Rabbit mAb (ABclonal, Cat. #A24422, 1:1000), phospho-tau-T181 rabbit mAb (ABclonal, Cat. #AP1387, 1:1000, Wuhan, China), and IGF2 (Signalway Antibody, Cat. #32592-2, 1:1000, Frederick, MA). The secondary antibody was Goat Anti-Rabbit IgG H&L (HRP) (Abcam, Cat. #ab205718, 1:5000, Cambridge, UK).

### ELISA for Aβ_40_

WT or *APOE*4/4 mouse brain tissues were homogenized in cold PBS containing a protease inhibitor cocktail and centrifuged at 15,000 × *g* for 20 min at 4 °C. Aβ_40_ levels were analyzed using a mouse Aβ_40_ ELISA kit (Cat. #ML00185996T, MIbio, Shanghai, China) according to the manufacturer’s instructions.

### Statistical analysis

GraphPad Prism 8.0 (GraphPad Software) was used for statistical analysis. All data are presented as the mean ± SD. For two-group comparisons, significance was assessed by Student’s *t* test. For multiple group comparisons, significance was assessed by one-way ANOVA with Tukey’s multiple comparison test. *P* < 0.05 was considered statistically significant.

## Results

### Early, multiple intravenous injection of *APOE*3/3-PCs rescues learning and memory decline in aged *APOE*4/4 mice

*APOE*3/3 hiPSCs were generated from PBMCs of two healthy donors using Sendai viral vectors and then induced to differentiate into pericytes (*APOE*3/3-PCs) of neural crest origin as previously reported [18, 19]. To investigate the potential therapeutic benefit of pericyte transplantation, a total of 1 × 10^6^ HDFs, *APOE*3/3-PCs, or an equal volume of PBS was intravenously injected to 18-month-old *APOE*4 homozygote mice. One month later, OFT and NOR were performed to evaluate cognition (Fig. S1a). However, no significant change was observed among the PBS, HDFs and PCs groups, with all *APOE*4/4 mice showing poor performance compared to age-matched WT mice (Fig. S1b–h). These findings suggest that a single transplantation of PCs may not rescue the cognitive decline in aged *APOE*4/4 mice, possibly because the pathological damage had already occurred and cannot be apparently rescued.

**Fig. 1:**
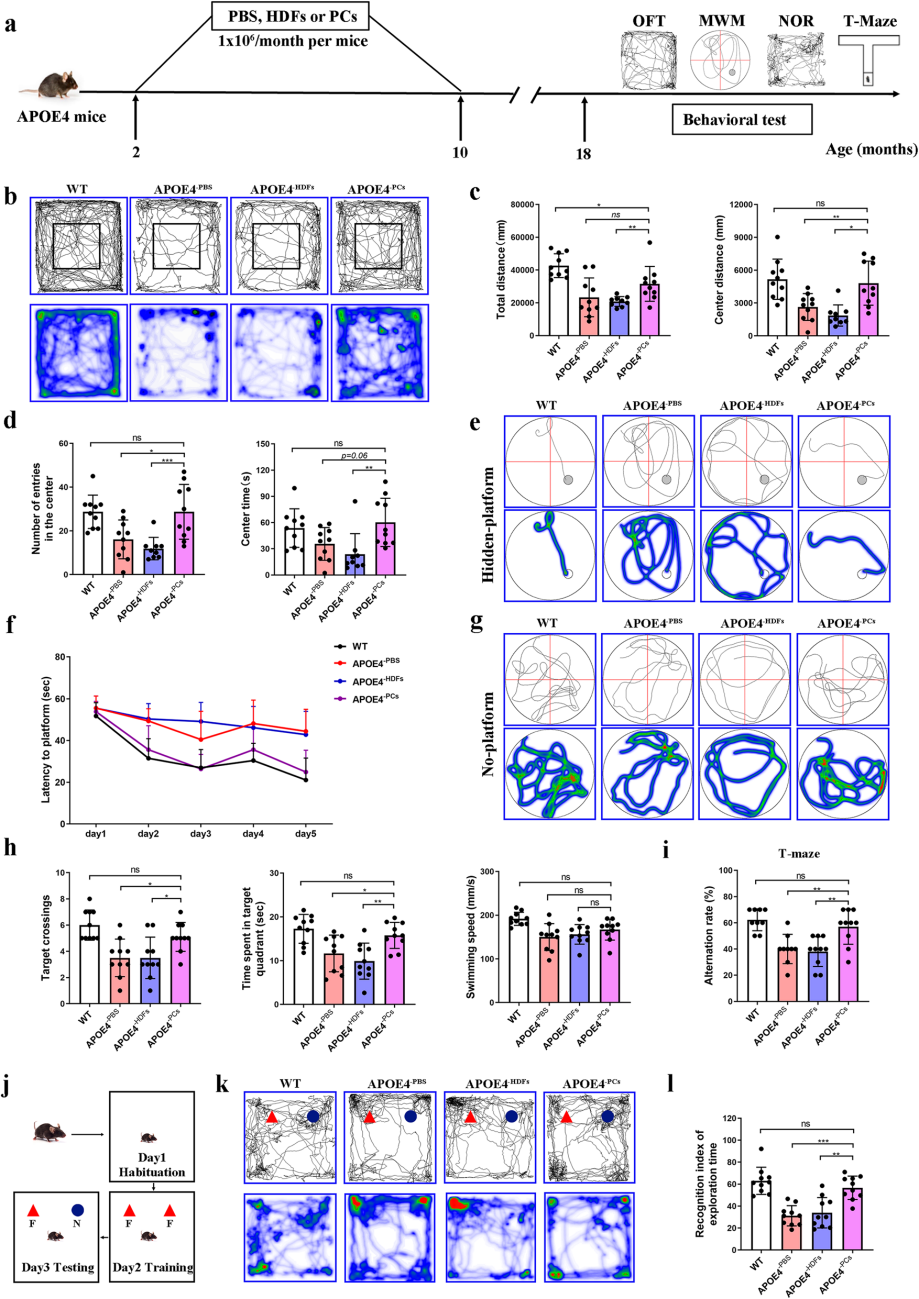
Early, multiple intravenous transplantation of *APOE*3/3-PCs rescued learning and memory decline in aged *APOE*4/4 mice. **a** Schematic illustrating the timeline of the study. **b**–**d** Mouse tracing images and heat maps (**b**), as well as behavioral analysis (**c**, **d**) in the open field test. **e**–**g** Swimming trajectories on day 5 with a hidden-platform (**e**), escape latencies from day 1 to day 5 (**f**), and swimming trajectories in the probing test on day 6 (**g**) in the Morris water maze (MWM). **h** Time spent and number of crossings in the target quadrant in the MWM. No difference was observed for the average swimming speed among PBS, HDF and PC groups. **i** T-Maze test results. **j** Schematic of the new object recognition (NOR) test (new object, N; familiar object, F). **k** Exploration trajectory of each group in the NOR test. **l** Recognition index of exploration in the NOR test. *n* = 9–10 mice per group, means ± SD. One-way ANOVA with Tukey’s multiple comparison test; ns, non-significant, *P < 0.05; **P < 0.01; ***P < 0.001

Since increased BBB damage was observed as early as 2–3 months of age in *APOE*4/4 mice [14], we next asked whether early and multiple transplantations can improve BBB function and cognition in *APOE*4/4 mice. Thus, mice were treated once a month for consecutive 8 months, starting at the age of 2–3 months. Behavioral tests were performed at 18 months (Fig. 1a). In the OFT test, WT mice and the PCs group mice had longer total distance travelled and longer distance travelled in the central area (Fig. 1b–c). However, mice in the PBS and HDFs groups showed little interest in entering the central field of the chamber as indicated by the number of entries in the center field or the center time (Fig. 1b–d). In the MWM test, during the training phase (hidden platform), mice treated with PCs had lower latency to find the platform compared to the PBS or HDFs controls (Fig. 1e–f). The mice in the PC group also spent more time in the target quadrant and had more target quadrant crossings in the probing test (no platform). However, no significant difference was found in the moving speed among the PBS, HDF, and PC groups (Fig. 1g, h). In the spontaneous alternation T-maze test, the PBS and HDF groups showed lower alternation rate, while PC transplantation markedly improved the alternation rate (Fig. 1i). The NOR test reflects the learning and memory ability of mice based on their natural tendency to explore novel objects instead of familiar ones when exposed to a novel environment. Our results indicated that PC transplantation significantly improved the recognition index of exploration time in the novel object, compared to PBS and HDF groups (Fig. 1j–l). To further validate our findings, we performed cell transplantation using pericytes derived from another *APOE*3/3 hiPSCs line and observed similar therapeutic effects in *APOE*4/4 mice (data not shown).

Altogether, these results demonstrate that early and multiple *APOE*3/3-PC transplantation improves cognition in aged *APOE*4/4 mice.

### *APOE*3/3-PC transplantation ameliorates AD pathologies in aged *APOE*4/4 mice

**Fig. 2:**
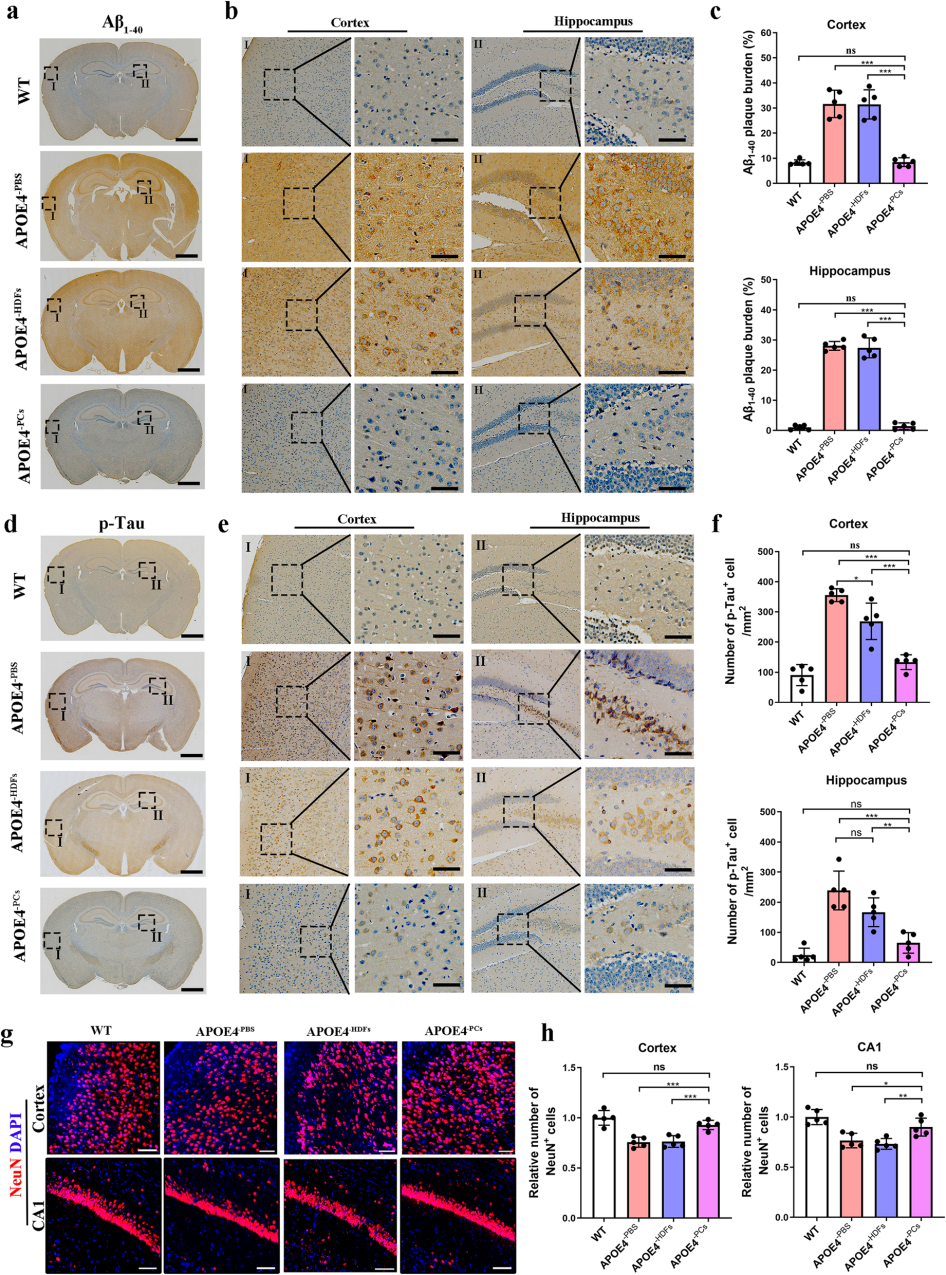
*APOE*3/3-PCs transplantation ameliorated AD pathologies in aged *APOE*4/4 mice. **a** Immunohistochemistry using anti-Aβ_1-40_ antibody. **b** Zoomed areas I (cortex) and II (hippocampus) from **a**. Scale bars, 1 mm (**a**) and 50 μm (**b**). **c** Statistics of Aβ burden (%) in the cortex and hippocampus. **d** Immunohistochemistry using anti-p-tau (Thr181) antibody. **e** Zoomed areas I (cortex) and II (hippocampus) from **d**. Scale bars, 1 mm (**d**) and 50 μm (**e**). **f** Number of p-tau^+^ cell per mm^2^ in the cortex and hippocampus. **g**, **h** Representative images (**g**) and quantification (**f**) of NeuN^+^ neurons in the cortex and CA1 area in the WT, PBS, HDF and PCs groups. Scale bars, 50 μm. *n* = 5 mice per group, means ± SD. One-way ANOVA with Tukey’s multiple comparison test; ns, non-significant, *P < 0.05; **P < 0.01; ***P < 0.001

To determine whether the improvement of cognition in aged *APOE*4/4 mice was accompanied by amelioration of AD pathology, we performed IHC to evaluate the levels of Aβ_1-40_ and p-tau. In the PBS treatment group, Aβ_1-40_ plaques were intensely stained and predominantly detected around neuronal cells in the cortex and the hippocampus of aged *APOE*4/4 mice (Fig. 2a–c). As expected, the Aβ_1-40_ plaque burden was significantly decreased in the PC group, which was almost comparable with the WT group (Fig. 2a–c). However, no significant change in Aβ_1-40_ plaque burden was detected in the HDFs group compared with the PBS group (Fig. 2a–c). Moreover, transplantation of PCs greatly reduced the number of p-tau^+^ cells per unit area (mm^2^) in the cortex and the hippocampus compared with the PBS group (Fig. 2d–f). We also noted mild p-tau improvement in the HDFs-treated mice versus the PBS group (Fig. 2d–f). These results were further confirmed by ELISA and western blotting analysis (Fig. S2a–d).

Neuron loss is a prominent pathological feature of AD, caused by excessive deposition of Aβ and p-tau [23]. We measured NeuN^+^ neuronal cells among the four groups, and found that the numbers of NeuN^+^ cells in the cortex and CA1 area were comparable between the HDFs group and the PBS group (Fig. 2g, h). In contrast, PCs transplantation significantly increased the number of NeuN^+^ cells compared with PBS or HDFs (Fig. 2g, h). Microglia, the innate immune cells of the central nervous system (CNS), play a multifaceted role in AD, contributing to neuroinflammation and neurodegeneration [24]. Increased number and activation of microglia have been observed in the brains of AD patients or mouse models [25]. Remarkably, our results revealed that transplantation of PCs not only significantly reduced the number of Iba1^+^ microglial cells, but also shifted the morphology of microglia toward the homeostatic form compared with PBS or HDFs (Fig. S2e, f).

In summary, these results demonstrated that early and multiple transplantation of *APOE*3/3-PCs can reduce Aβ deposition, p-tau accumulation, neuron loss and microglial activation in aged *APOE*4/4 mice.

### *APOE*3/3-PCs preserve BBB integrity in *APOE*4/4 mice

In our previous study, we demonstrated that intravenous injection of hiPSCs-CNC PCs could efficiently restore BBB properties in the tMCAO mouse model [18]. Thus, we evaluated the changes of BBB permeability in *APOE*4/4 mice after *APOE*3/3-PC transplantation.

**Fig. 3:**
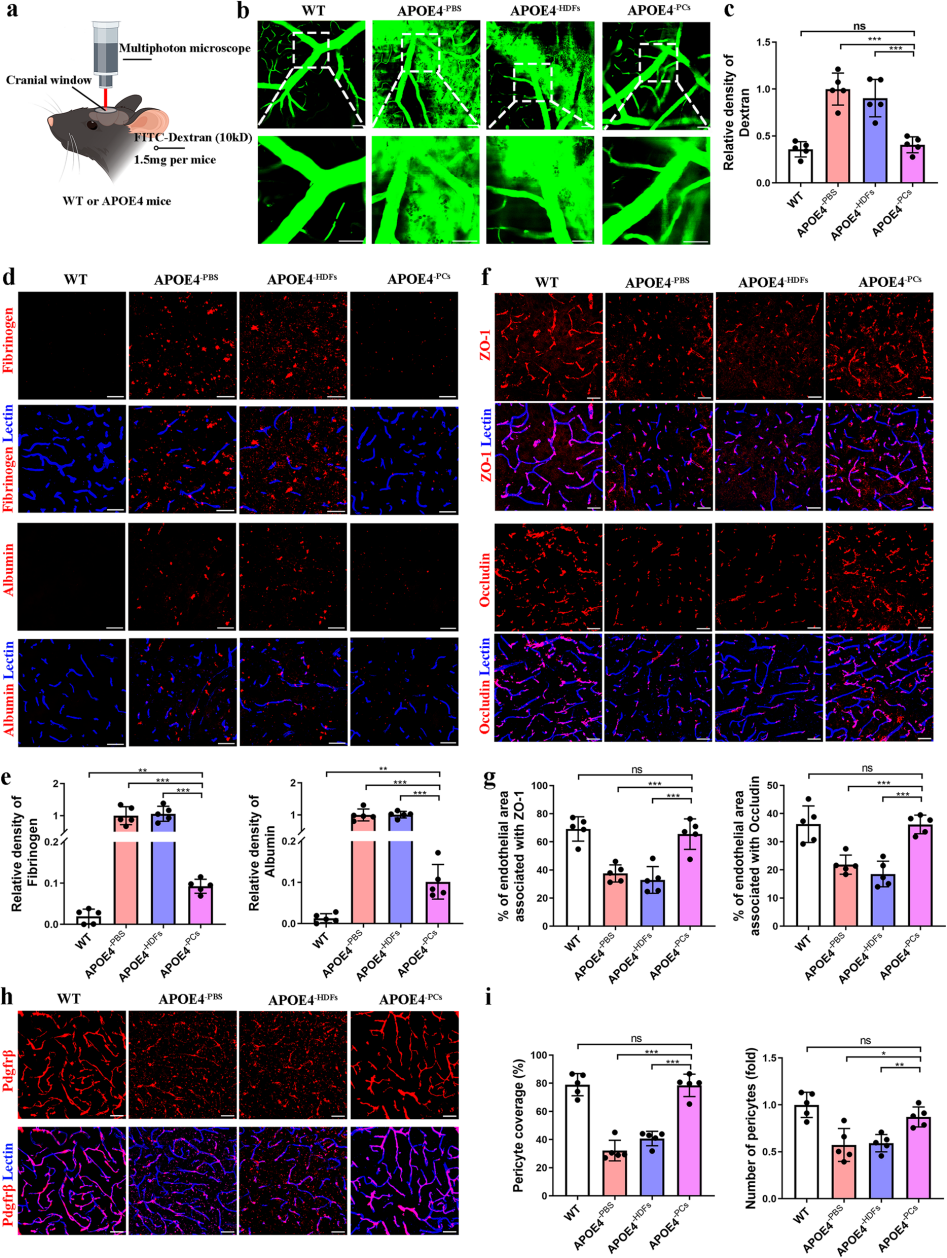
*APOE*3/3-PC transplantation preserved BBB integrity in *APOE*4/4 mice. **a** Design for dextran leakage detection in vivo using multiphoton microscopy. **b** Images of FITC-dextran leakage in WT, PBS, HDF and PC mice in vivo. Scale bar, 50 μm (upper), 20 μm (lower). **c** Relative density of dextran. **d**, **e** Representative confocal images and quantification of fibrinogen and albumin leakage around lectin-labeled capillaries. Scale bars, 50 μm. **f**, **g** Confocal images and quantification of lectin-labeled capillaries covered by tight junction proteins ZO1 and Occludin. Scale bars, 50 μm. **h** Immunofluorescence staining of pdgfrβ^+^ pericytes in mouse brain. Scale bars, 50 μm. **i** Quantification of the percentage of Lectin^+^ capillaries covered by pdgfrβ^+^ pericytes and the relative number of pdgfrβ^+^ pericytes. All data are shown as means ± SD. *n* = 5 mice per group, one-way ANOVA with Tukey’s multiple comparison test; ns, non-significant, *P < 0.05; **P < 0.01; ***P < 0.001

In this study, we used multiphoton microscopy to observe vascular leakage in vivo after intravenously injecting FITC-conjugated dextran at a molecular weight of 4kD (Fig. 3a). We observed an intact vascular outline in the WT and PC groups, while significant dextran leakage was observed in the PBS and HDF groups (Fig. 3b, c). Extravascular deposition of fibrinogen and albumin are commonly used as markers for BBB integrity [26]. As expected, PC transplantation, instead of PBS or HDFs treatment, effectively reduced fibrinogen/albumin deposits (Fig. 3d, e).

Compared to WT mice, *APOE*4/4 mice exhibited a moderate loss of capillary density in the cortex and hippocampus, which was well preserved following PC transplantation (Fig. S3a, b). Next, we evaluated the integrity of BBB using antibodies against tight junction proteins ZO1 and Occludin, which are associated with BBB breakdown in *APOE*4/4 mice [27, 28]. Immunofluorescence staining indicated a significant loss of ZO1 and Occludin in the PBS group and no obvious improvement after HDFs transplantation, while PCs drastically protected ZO1 and Occludin from degradation (Fig. 3f, g). Given the role of perivascular pericytes in the maintenance of the BBB, we asked whether PC transplantation could prevent pericyte loss in *APOE*4/4 mice. To our surprise, mice in the PC and WT groups displayed similar percentages of pericyte coverage and comparable numbers of Pdgfrβ^+^ pericytes, while dramatic loss of pericyte coverage and decreased cell number were detected in the PBS and HDF groups (Fig. 3h, i). Furthermore, we observed a significant reduction in CD206^+^ perivascular macrophages in *APOE*4/4 mice, which was partially rescued by PC transplantation (Fig. S3c, d). GFAP staining revealed a significant decrease in GFAP^+^ endfeet coverage on blood vessels in the PBS and HDF groups, while PC transplantation partially increased the GFAP^+^ endfeet coverage (Fig. S3e, f). Besides, the number of GFAP^+^ astrocytes in the cortical region was significantly increased in the PBS and HDF groups, while a partial decrease was observed in the PCs transplantation group (Fig. S3g).

Altogether, these results demonstrated that transplantation of *APOE*3/3-PCs could preserve BBB integrity and reverse intrinsic pericyte loss in *APOE*4/4 mice.

### ApoVs generated from the transplanted *APOE*3/3-PCs were phagocytosed by endogenous pericytes in *APOE*4/4 mice

**Fig. 4:**
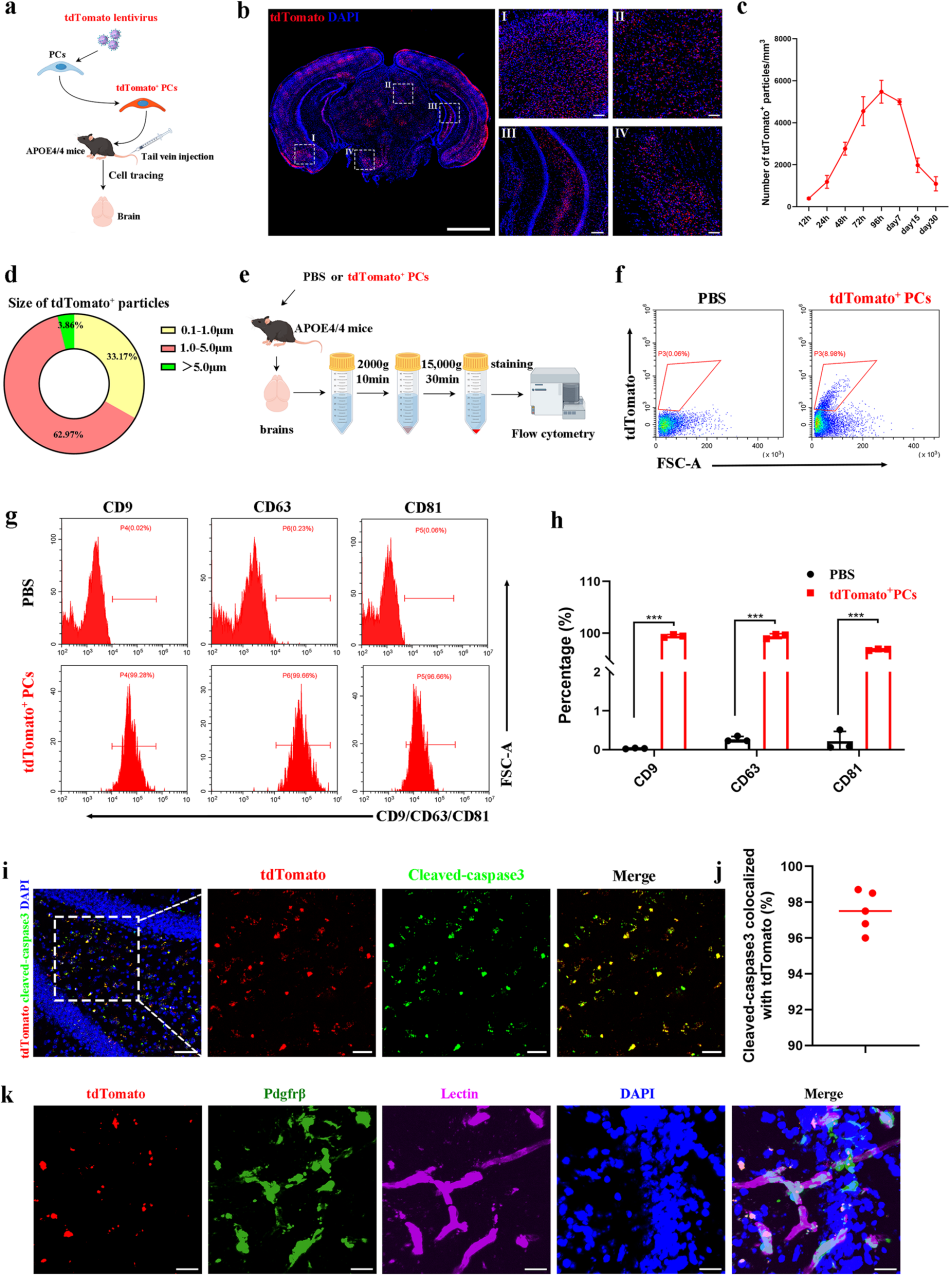
ApoVs were generated from *APOE*3/3-PCs and phagocytosed by endogenous pericytes in *APOE*4/4 mice*.* **a** Strategies to generate tdTomato^+^ PCs for cell tracing. **b** Representative images showing distribution of tdTomato signals in *APOE*4/4 mouse brain at 3 days post-transplantation. Scale bar, 200 μm. Zoomed areas of cortex (I), superior colliculus (II), hippocampus (III) and hypothalamus (IV). Scale bars, 50 μm. **c** Number of tdTomato^+^ signals per mm^2^ in the cortex at different time points. **d** Size analysis of tdTomato^+^ signals in the cortex at 3 days post-transplantation. **e** Strategies to harvest tdTomato^+^ particles from *APOE*4/4 mouse brain. **f** Representative images showing the percentage of tdTomato^+^ particles by flow cytometry. **g**, **h** Flow cytometry analysis for the expression of CD9, CD63 and CD81 on tdTomato^+^ particles. **i, j** Confocal microscopy images (**i**) and quantification analysis (**j**) of cleaved-caspase3 signals colocalized with tdTomato^+^ signals at 3 days post-transplantation. Scale bars, 50 μm. **k** Confocal images showing that tdTomato^+^ ApoVs were engulfed by pdgfrβ^+^ pericytes in *APOE*4/4 mice. Scale bars, 50 μm. Student’s *t* test was used for comparisons between two groups; ns, non-significant, *P < 0.05; **P < 0.01; ***P < 0.001

To verify the distribution and characteristics of the *APOE*3/3-PCs in vivo, we labeled *APOE*3/3-PCs with tdTomato^+^ lentivirus (Fig. 4a). According to previous experimental findings, intravenously administered cells, such as mesenchymal stem cells (MSCs), are predominantly distributed in the lungs, liver, and spleen [29]. Thus, distribution of transplanted cells in the lungs, liver, and spleen of *APOE*4/4 mice was evaluated on days 7, 15, and 30 after transplantation. In the lungs, abundant structurally intact tdTomato^+^ pericytes were observed on day 7, but these cells were reduced on day 15 and almost disappeared on day 30, suggesting progressive clearance (Fig. S4). In the liver and spleen, both intact tdTomato^+^ cells and small tdTomato^+^ signals were present on day 7. On day 15, the intact cells markedly declined with a concomitant increase in small tdTomato^+^ signals. The intact transplanted cells were rarely observed on day 30, demonstrating effective clearance of the donor cell population (Fig. S4). However, intact tdTomato^+^ *APOE*3/3-PCs were rarely observed in the brains of *APOE*4/4 mice 3 days after transplantation. Intriguingly, we observed extensive tdTomato^+^ signals in the whole brain area, including cortex (I), superior colliculus (II), hippocampus (III) and hypothalamus (IV) (Fig. 4b). Next, we harvested brain samples at different time points after tail vein injection of tdTomato^+^ PCs. We observed increased number of tdTomato^+^ signals from 12 h, which reached a peak at ~ 96 h. Strikingly, even after 30 days of cell transplantation, the tdTomato^+^ signals could still be detected in the brains of *APOE*4/4 mice (Fig. 4c and Fig. S5a). In addition, the majority (62.97%) of tdTomato^+^ signals had a size of 1–5 μm (Fig. 4d), implying that the tdTomato^+^ signals might be a kind of extracellular vesicle (EV). To confirm this hypothesis, we conducted an mCherry niche-labelling system in which PCs co-express the sLP–mCherry and EGFP (Fig. S5b). sLP–mCherry is a secreted fluorescent mCherry protein containing a modified lipid-permeable transactivator of transcription peptide, which was used to label EVs [21]. Using this system, we observed EGFP in the intracellular space covered by mCherry signal (Fig. S5c). Further, we tried to harvest tdTomato^+^ signals from mouse brain homogenate by a series of centrifugation operations (Fig. 4e, f). We found that almost all tdTomato^+^ signals expressed EV markers CD9, CD63 and CD81 (Fig. 4g, h). Furthermore, IF staining revealed that almost all tdTomato^+^ signals were co-localized with cleaved-caspase 3, a typical apoptosis flag (Fig. 4i, j). Thus, we considered that most of the tdTomato^+^ signals arise from ApoVs.

Next, we tried to trace the source of tdTomato^+^ ApoVs. Given that the majority of transplanted pericytes accumulated in the lungs of *APOE*4/4 mice, we subsequently performed further analyses of the pulmonary tissue and observed that some tdTomato^+^ cells exhibited signs of lysis and vesicle formation (Fig. S5d). To further confirm that the tdTomato^+^ ApoVs were released from the lungs, we harvested lung tissues from *APOE*4/4 mice on day 3 after cell injection and observed real-time ApoV release ex-vivo with multiphoton microscopy. We found time-dependent release of ApoVs from mouse lungs after pericyte transplantation (Fig. S5e, f). Due to their small size (1–5 μm), ApoVs can readily pass through pulmonary capillaries (8–10 μm diameter), enter systemic circulation, and reach the mouse brain [30]. Furthermore, we asked which cell type can uptake the PCs-derived ApoVs in *APOE*4/4 mice brain. We found that the tdTomato^+^ ApoVs were engulfed by pericytes, neurons and microglia, but not by astrocytes (Fig. 4k and Fig. S5g).

In summary, we demonstrated that transplanted *APOE*3/3-PCs generate huge numbers of ApoVs in the lung or other tissues in vivo, which could circulate to the mouse brain and be engulfed by multiple cell types including pericytes in the brains of *APOE*4/4 mice.

### *APOE*3/3-PCs-derived ApoVs improve physiological functions of *APOE*4/4-PCs in vitro

We observed increased pdgfrβ^+^ pericyte number and pericyte coverage in *APOE*4/4 mice after PCs transplantation (Fig. 3h, i). Additionally, the transplanted *APOE*3/3-PCs released huge numbers of ApoVs, which were then engulfed by endogenous pericytes (Fig. 4k). Thus, we inferred that ApoVs engulfed by pericytes may prevent *APOE4*-related pericyte degeneration.

**Fig. 5:**
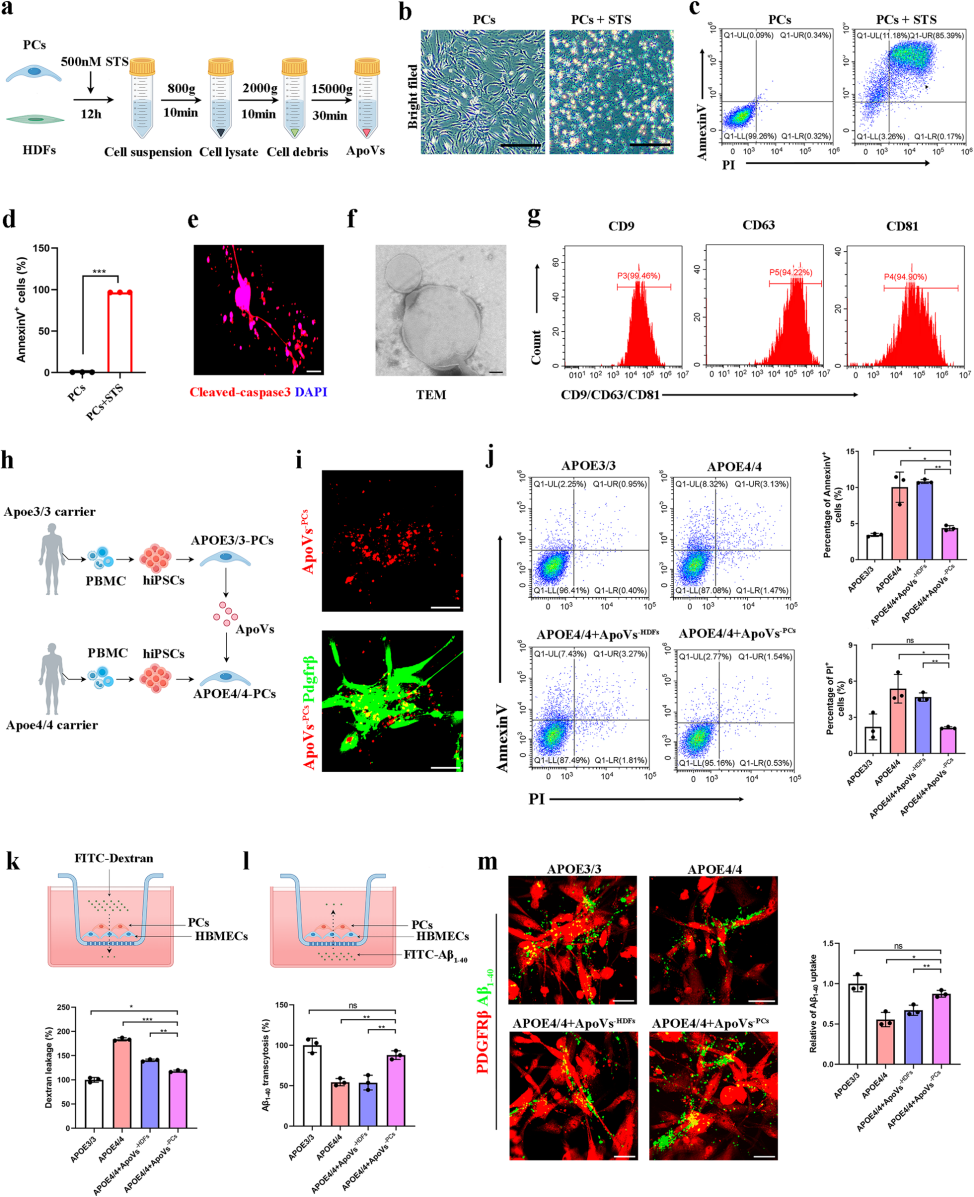
*APOE*3/3-PCs-derived ApoVs improved physiological functions of *APOE*4/4-PCs in vitro. **a** Illustration of ApoV production and collection. *APOE*3/3-PCs or HDFs were treated with 500 nmol/L STS for 12 h and apoptotic cell suspensions were isolated using a sequential centrifugation system. **b** Morphological change of *APOE*3/3-PCs before and after 12 h treatment with STS. Scale bar, 50 μm. **c**, **d** Flow cytometry analysis and quantification of *APOE*3/3-PCs apoptosis rate using AnnexinV/PI staining after STS induction (*n* = 3 biological repeats for each group). **e** Cleaved-caspase 3 assay showing the apoptotic cells or ApoVs after STS induction. Scale bar, 30 μm. **f** TEM image of the morphology of ApoVs derived from *APOE*3/3-PCs. Scale bar, 100 nm. **g** Flow cytometry analysis showing expression of CD9, CD63 and CD81 on the surface of *APOE*3/3-PC-derived ApoVs. **h** Schematic of the generation of *APOE*3/3-PCs and *APOE*4/4-PCs from hiPSCs for in vitro studies. **i** ApoVs derived from *APOE*3/3-PCs were engulfed by *APOE*4/4-PCs in vitro. Scale bars, 50 μm. **j** AnnexinV/PI staining showing the cell death rate in *APOE*3/3-PCs, *APOE*4/4-PCs, *APOE*4/4-PCs + ApoVs^−HDFs^ and *APOE*4/4-PCs + ApoVs^−PCs^ groups (*n* = 3 biological repeats for each group). **k** Dextran leakage assay was performed in *APOE*3/3-PCs, *APOE*4/4-PCs, *APOE*4/4-PCs + ApoVs^−HDFs^ and *APOE*4/4-PCs + ApoVs^−PCs^ groups (*n* = 3 biological repeats for each group). **l** Aβ transcytosis assay was performed in *APOE*3/3-PCs, *APOE*4/4-PCs, *APOE*4/4-PCs + ApoVs^−HDFs^ and *APOE*4/4-PCs + ApoVs^−PCs^ groups (*n* = 3 biological repeats for each group). **m** Representative images of Aβ_1-40_ uptake and statistical result between the *APOE*3/3-PCs, *APOE*4/4-PCs, *APOE*4/4-PCs + ApoVs^−HDFs^ and *APOE*4/4-PCs + ApoVs^−PCs^ groups. Scale bars, 50 μm (*n* = 3 biological repeats for each group). One-way ANOVA with Tukey’s multiple comparison test; ns, non-significant, *P < 0.05; **P < 0.01; ***P < 0.001

To directly test this hypothesis, we used STS to induce apoptosis of cultured *APOE*3/3-PCs or HDFs, and ApoVs generated from these cells were collected by a sequential centrifugation system (Fig. 5a). *APOE*3/3-PCs showed significant morphological alterations after 12 h of STS induction (Fig. 5b). Apoptotic condition was verified by the Apoptosis Detection Kit and cleaved-caspase3 staining (Fig. 5c–e). TEM confirmed the morphology of induced ApoVs (Fig. 5f). Flow cytometry revealed robust expression of extracellular surface markers CD9, CD63 and CD81 in ApoVs (Fig. 5g). Then the *APOE*4/4 hiPSCs were established, characterized (Fig. S6), and differentiated into pericyte-like cells (Fig. 5h). The ApoVs^−PCs^ were added into the culture medium of *APOE*4/4-PCs. The ApoVs^−PCs^ could be engulfed by *APOE*4/4-PCs in vitro (Fig. 5i). Compared with *APOE*3/3-PCs, we observed increased cell death of *APOE*4/4-PCs by Annexin V/PI staining. Nonetheless, when co-cultured with ApoVs^−PCs^ for 48 h, the cell death rate of *APOE*4/4-PCs was substantially rescued (Fig. 5j).

BBB restricts neurotoxic plasma components from entering the brain and regulates the removal of metabolic waste products from the brain to the systemic circulation, including Aβ [31, 32]. We then first evaluated BBB stability by co-culture of HBMECs with *APOE*3/3- or *APOE*4/4-PCs on transwell inserts followed by addition of FITC-Dextran in the upper well. The *APOE*4/4-PC group showed increased dextran leakage, while ApoVs^−PCs^, rather than ApoVs derived from HDFs (ApoVs^−HDFs^), significantly reduced the permeability in the *APOE*4/4-PCs co-culture system (Fig. 5k). Aβ transport by pericytes is one of the primary mechanisms for Aβ clearance across the brain to the blood [33]. We found weakening of the transport capacity of *APOE*4/4-PCs, which was significantly improved by ApoVs^−PCs^ treatment (Fig. 5l). Since pericytes have been shown to capture Aβ through LRP1 (low-density lipoprotein receptor related protein-1) [34, 35], PCs were co-cultured with FITC-labeled Aβ_1-40_ for 48 h. We found more Aβ_1-40_ attachment or swallowing by *APOE*3/3-PCs than by *APOE*4/4-PCs, and ApoVs^−PCs^ treatment greatly improved the capture capacity of *APOE*4/4-PCs for Aβ_1-40_ (Fig. 5m).

To further validate that the therapeutic effect is uniquely attributable to ApoVs, we cultured *APOE*4/4-PCs with same quantity of exosomes or ApoVs from *APOE*3/3-PCs. Our results demonstrated that ApoVs, but not exosomes, could rescue *APOE*4/4-PCs viability and improve the pseudo-BBB integrity (Fig. S7a–d).

Altogether, we successfully generated ApoVs from *APOE*3/3^-PCs^ by STS induction and found ApoVs^−PCs^ treatment could promote functional recovery of *APOE*4/4-PCs in vitro.

### IGF2 in ApoVs plays a vital role in functional recovery of *APOE*4/4-PCs in vitro

**Fig. 6:**
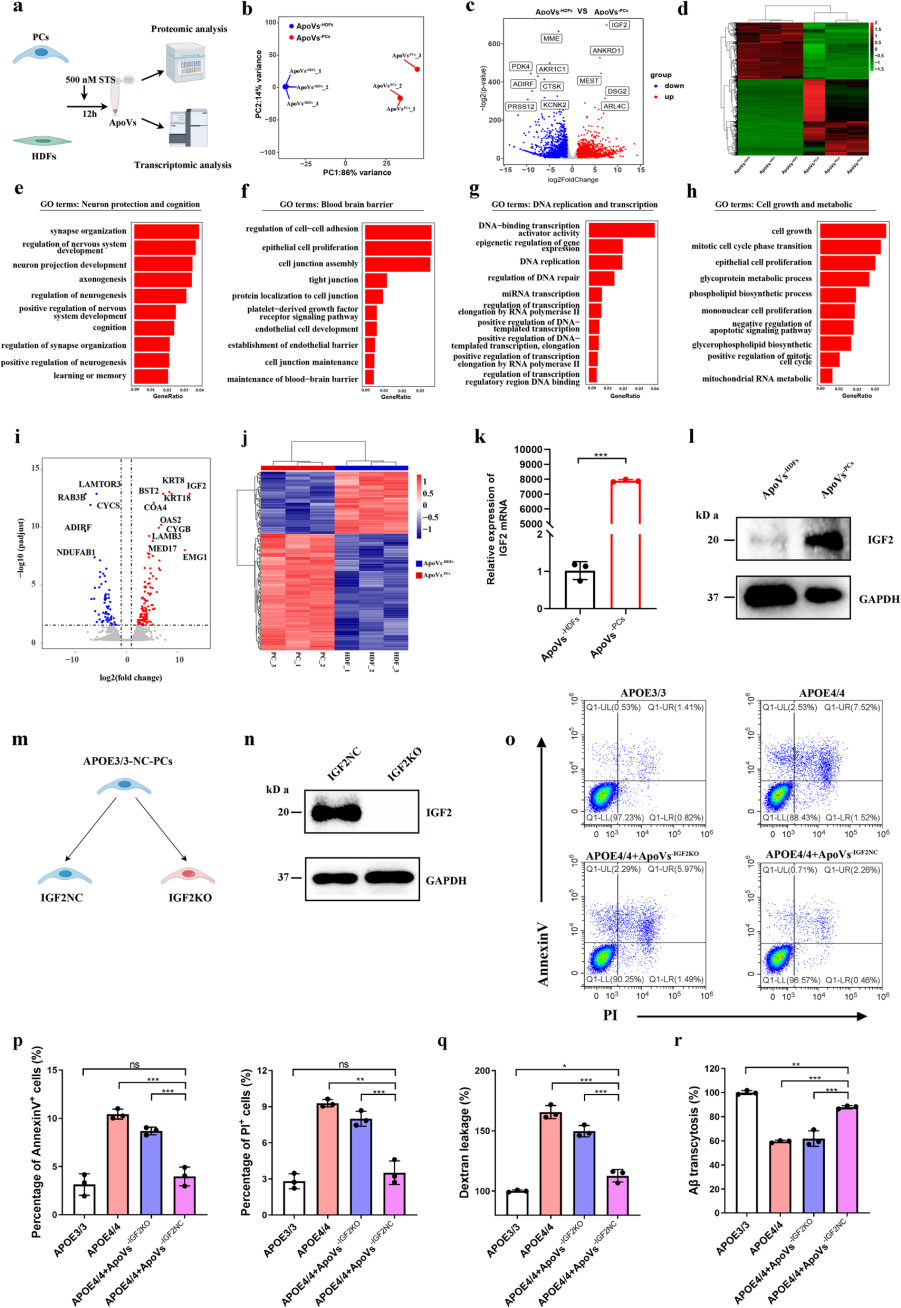
IGF2 in ApoVs plays a vital role in functional recovery of *APOE*4/4-PCs in vitro. **a** ApoVs derived from *APOE*3/3-PCs or HDFs were subjected to transcriptomic analysis and proteomic analysis. **b** Principal component analysis (PCA) evaluating the similarities of gene expression profiles between ApoVs^−HDFs^ and ApoVs^−PCs^. **c, d** Volcano plot and heatmap of differential gene expression analysis between ApoVs^−HDFs^ and ApoVs^−PCs^. **e**–**h** The differentially expressed genes were subjected to GO enrichment analysis. The GO terms related to neuron protection and cognition (**e**), blood–brain barrier maintenance (**f**), DNA replication and transcription (**g**) and cell growth and metabolic (**h**) were significantly enriched in ApoVs^−PCs^. **i, j** Volcano plot (**i**) and heatmap (**j**) of protein expression between ApoVs^−HDFs^ and ApoVs^−PCs^. **k, l** qPCR and western blot analysis of IGF2 mRNA expression and protein level in ApoVs^−HDFs^ and ApoVs^−PCs^. **m, n** IGF2 knockout in *APOE*3/3-PCs using the CRISPR-Cas9 system. IGF2 expression was confirmed by western blot analysis. **o, p** Flow cytometry analysis with annexin V and PI staining revealed cell death rate in *APOE*3/3-PCs, *APOE*4/4-PCs, *APOE*4/4-PCs + ApoVs^−PCs−IGF2KO^ and *APOE*4/4-PCs + ApoVs^−PCs−IGF2NC^ groups (*n* = 3 biological repeats for each group). **q** Representative dextran leakage in *APOE*3/3-PCs, *APOE*4/4-PCs, *APOE*4/4-PCs + ApoVs^−PCs−IGF2KO^ and *APOE*4/4-PCs + ApoVs^−PCs−IGF2NC^ groups (*n* = 3 biological repeats for each group). **r** Representative Aβ transcytosis in *APOE*3/3-PCs, *APOE*4/4-PCs, *APOE*4/4-PCs + ApoVs^−PCs−IGF2KO^ and *APOE*4/4-PCs + ApoVs^−PCs−IGF2NC^ groups (*n* = 3 biological repeats for each group). One-way ANOVA with Tukey’s multiple comparison test; ns, non-significant, *P < 0.05; **P < 0.01; ***P < 0.001

To further explore the underlying mechanism of ApoVs^−PCs^, we performed transcriptomic and proteomic analysis of ApoVs^−HDFs^ and ApoVs^−PCs^ (Fig. 6a). RNA-sequencing results showed that 3449 genes were upregulated and 2541 genes were downregulated in ApoVs^−PCs^ compared with ApoVs^−HDFs^ (Fig. 6b–d). *IGF2* was one of the most significantly different mRNAs between ApoVs^−HDFs^ and ApoVs^−PCs^ (log_2_FoldChange = 7.53, − log_2_(*P*-value) > 600) (Fig. 6c). Next, the differentially expressed genes were subjected to gene ontology (GO) enrichment analysis. A total of 722 GO terms in biological process, 29 GO terms in cellular component and 60 GO terms in molecular function were enriched in ApoVs^−PCs^, including GO terms related to neuron protection and cognition, blood brain barrier maintenance, DNA replication and transcription, and cell growth and metabolic (Fig. 6e–h). In proteomic analysis, we observed a total of 103 proteins, including IGF2 (log_2_FoldChange = 12.8), were expressed at markedly higher levels in ApoVs^−PCs^ compared to ApoVs^−HDFs^ (Fig. 6i, j). RT–qPCR and western blot analysis revealed that IGF2 was indeed highly enriched in ApoVs^−PCs^ (Fig. 6k, l). In addition, IGF2 was most abundantly expressed in ApoVs^−PCs^ and lowest in exosomes^−PCs^ (Fig. S7e). Furthermore, *IGF2* mRNA was specifically and significantly enriched during the generation of ApoVs (Fig. S7f).

We then focused on whether IGF2 is involved in the reparative effects of ApoVs^−PCs^. We constructed *IGF2*-knockout *APOE*3/3-PCs using CRISPR-Cas9 system, which was confirmed by western blot analysis (Fig. 6m, n and Fig. S8a). Then, ApoVs of *IGF2*-knockout *APOE*3/3-PCs (ApoVs^−PCs−IGF2KO^) and non-targeting control *APOE*3/3-PCs (ApoVs^−PCs−IGF2NC^) were generated as described above, and cultured with *APOE*4/4-PCs. We found that *IGF2* deletion largely abolished the therapeutic effects of ApoVs^−PCs^, as exhibited by increased cell death, dextran transcytosis, and reduced Aβ transport ability (Fig. 6o–r). Besides, we observed dramatic cell death and function weakness of *APOE*3/3-PCs when *IGF2* was knocked out (Fig. S8b–e).

Together, the ApoVs^−PCs^ contain higher levels of IGF2 protein and mRNA compared with ApoVs^−HDFs^, which may play vital roles in the protective effect of ApoVs^−PCs^.

### ApoVs from *APOE*3/3-PCs achieved significant therapeutic effects in *APOE*4/4 mice

**Fig. 7:**
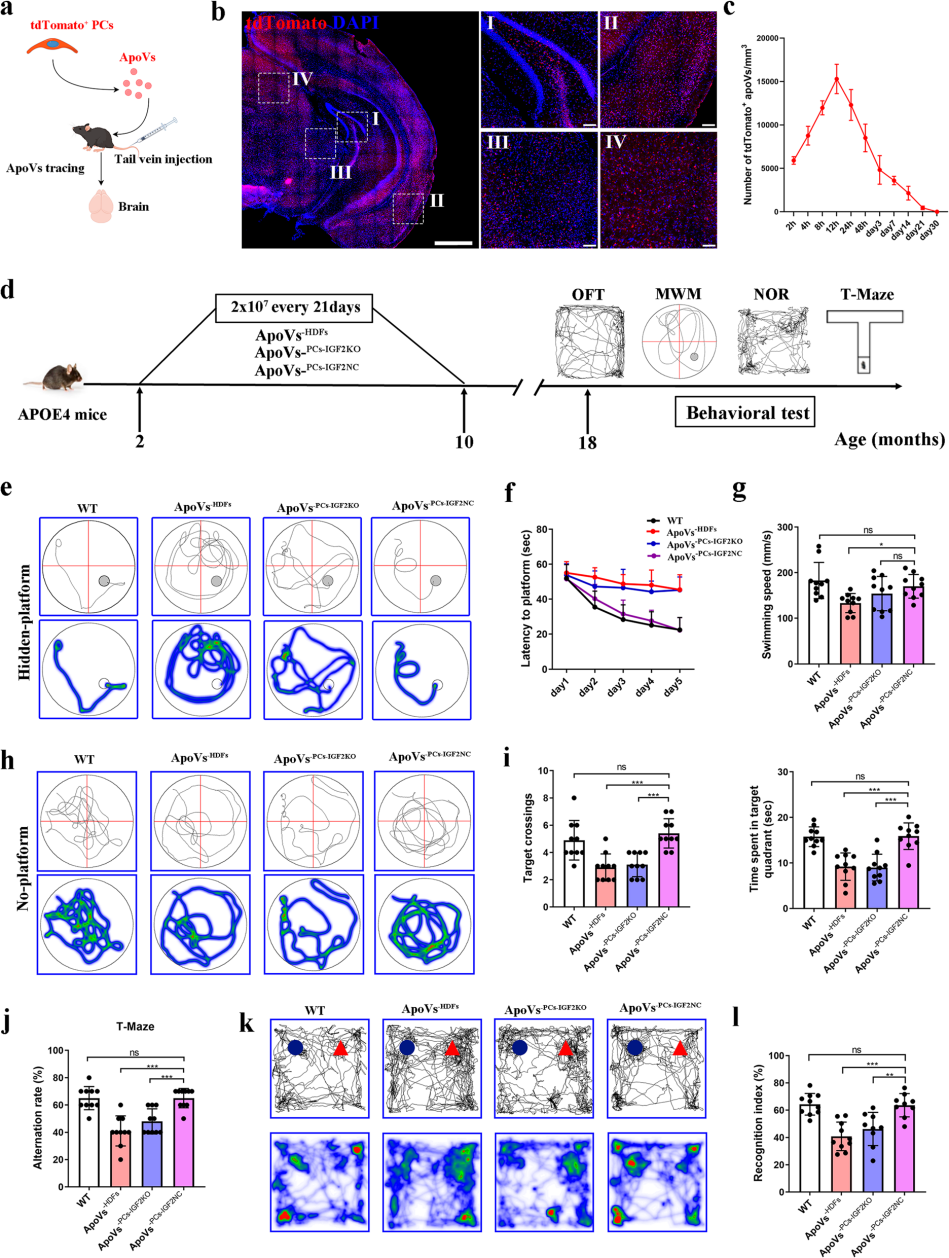
ApoVs from *APOE*3/3-PCs achieved significant therapeutic effects in *APOE*4/4 mice. **a** Strategies to generate tdTomato^+^ ApoVs for in vivo tracing. **b** Representative images showing distribution of tdTomato^+^-labeled ApoVs in *APOE*4/4 mouse brain at 48 h post-transplantation. Scale bar, 100 μm (left), 50 μm (right). **c** Number of tdTomato^+^ ApoVs per mm^2^ in the cortex at different time points. **d** Timeline of experiments. **e**–**i** Results of Morris water maze test, including swimming trajectories on day 5 with a hidden platform (**e**), latencies to find the platform on days 1–5 (**f**), swimming speed (**g**), swimming trajectories on day 6 in the probing trial (**h**), and the time spent and number of crossings in the target quadrant (**i**). **j** Results of the T-maze. **k** Exploration trajectory and heat maps in the new object recognition (NOR) test. New object, blue; familiar object, red. **l** Recognition index of exploration. All data are shown as means ± SD. *n* = 9–10 mice per group. One-way ANOVA with Tukey’s multiple comparison test; ns, non-significant, *P < 0.05; **P < 0.01; ***P < 0.001

ApoVs have shown therapeutic potential in animal studies [36, 37]. Thus, we asked whether transplantation of ex-vivo generated ApoVs from *APOE*3/3-PCs could produce similar curative effects as *APOE*3/3-PCs in *APOE*4/4 mice, and whether IGF2 is involved in the effects of *APOE*3/3-PCs in vivo. We injected 2 × 10^7^ tdTomato-labeled ApoVs intravenously into *APOE*4/4 mice, and mouse brains were collected to analyze the distribution of ApoVs throughout the brain from 2 h to day 30 after the infusion (Fig. 7a–c and Fig. S9a). The number of tdTomato^+^ ApoVs reached a peak at 12 h after ApoV transplantation and persisted for 21 days, but was not detected 30 days after transplantation. Based on this finding, *APOE*4/4 mice of 2–3 months were treated with 2 × 10^7^ ApoVs every 21 days for a total of 10 times (Fig. 7d).

At the end of experiment, MWM, NOR, T-maze, and OFT were performed to evaluate cognition recovery of *APOE*4/4 mice at 18 months old. In the WMW test, the latency to the platform was shorter in the ApoVs^−PCs−IGF2NC^ group compared with the ApoVs^−IGF2KO^ group on day 5 of training, with no difference between the ApoVs^−HDFs^ and the ApoVs^−PCs−IGF2KO^ groups (Fig. 7e, f). In the probing test, the number of crossings and the total time spent in the target quadrant were higher in the ApoVs^−PCs−IGF2NC^ group than in the ApoVs^−PCs−IGF2KO^ group, and comparable between ApoVs^−HDFs^ and ApoVs^−PCs−IGF2KO^ groups (Fig. 7h, i). Similarly, the ApoVs^−PCs−IGF2NC^ group showed better performance compared with the ApoVs^−IGF2KO^ group, while ApoVs^−HDFs^ and ApoVs^−IGF2KO^ groups showed no significant difference in the performance in T-maze test, NOR test, or the OFT (Fig. 7j–l and Fig. S9b, c). Taken together, these data indicate that transplantation of ApoVs derived from *APOE*3/3-PCs improved cognitive function in *APOE*4/4 mice, whereas IGF2 depletion largely abolished the treatment effect of ApoVs. This suggests an important role of IGF2 in the therapeutic capacity of ApoVs in *APOE*4/4 mice.

We further explored whether the *APOE*3/3-PCs-derived ApoVs can ameliorate AD pathologies and improve BBB function in *APOE*4/4 mice. The ApoVs^−PCs−IGF2NC^ group showed less Aβ and p-tau compared with the ApoVs^−HDFs^ group, while IGF2 depletion largely weakened this effect (Fig. S10a, b). In addition, the numbers of NeuN^+^ cells in the cortex and CA1 region were higher in the ApoVs^−PCs−IGF2NC^ group compared with the ApoVs^−HDFs^ group, while no increase of NeuN^+^ cells was observed in the ApoVs^−PCs−IGF2KO^ group (Fig. S10c, d). Next, BBB permeability was evaluated by immunostaining. We observed significant recovery of BBB integrity in ApoVs^−PCs−IGF2NC^, but not in the ApoVs^−HDFs^ or ApoVs^−PCs−IGF2KO^ group (Fig. S11a, b). The level of tight junction protein ZO-1 was drastically improved in the ApoVs^−PCs−IGF2NC^ group (Fig. S11c). ApoVs^−IGF2NC^ also prevented pericyte loss in *APOE*4/4 mice (Fig. S11d).

In summary, transplantation of ApoVs generated from *APOE*3/3-PCs achieved significant therapeutic effects in *APOE*4/4 mice, but these effects were reversed by *IGF2 *knockout.

## Discussion

In the past several decades, mice bearing genetic mutations linked to autosomal-dominant AD (ADAD), such as 5 × FAD mice, were widely used for AD-related research and therapy development. However, ADAD is estimated to account for only ~ 1% of AD cases [38, 39]. On the contrary, late-onset AD (LOAD) accounts for more than 95% of AD cases [40]. The *APOE* ε4 allele is the strongest genetic risk factor for LOAD, with three-to-four-fold increased risk for heterozygotes and a nearly 12-fold increased risk for homozygotes compared to the *APOE3* homozygotes, the most common genotype [41, 42]. *APOE*4/4 mice exhibit cognitive deficits at 15 months or older [43]. Here, we observed increased BBB leakage and pericyte degeneration in 2-month-old *APOE*4/4 mice (data not shown), and impaired behavior performance accompanied by AD typical pathologies (including abnormal deposition of Aβ and p-tau, neuron loss and microglial activation) in 18-month-old *APOE*4/4 mice. These results highlight the possibility that *APOE4*-induced pericyte/BBB impairment is a driver of LOAD. Indeed, it has been reported that *APOE4* leads to BBB breakdown by activating the CypA–NFκB–MMP9 pathway in pericytes, which in turn initiates neurodegenerative changes. Suppressing this pathway could reverse BBB breakdown and neuronal dysfunction [14]. Another study has demonstrated that the calcineurin nuclear factor of activated T cells (NFAT) signaling is selectively dysregulated in *APOE4* pericytes, which further induces cerebral amyloid angiopathy (CAA). Inhibition of the calcineurin–NFAT signaling reduces *APOE4*-associated CAA pathology in vitro and in vivo [44]. Collectively, the above evidence suggests that pericyte/BBB impairment is a potential therapeutic target for LOAD.

Brain pericytes, originating from neural crest during development, regulate blood flow, BBB stability and inflammation, and promote long-term memory [15, 45, 46]. Tachibana et al. reported that implantation of pericytes differentiated from mouse fibroblasts reduced AD pathology in APP/PS1 mice [16]. Thus, pericyte transplantation may have the potential to rescue AD-related cognitive deficits in *APOE*4/4 mice. Previous studies reported that a single dose transplantation of human embryonic stem cell (hESC)-derived MSCs or hESC-derived immunity-and-matrix regulatory cells can improve cognition of AD animals [47, 48]. We first tested whether a single injection of *APOE*3/3-PCs can rescue memory decline in aged *APOE*4/4 mice. However, no significant behavior change was observed between PBS, HDF and PC groups. Since AD pathologies in humans develop slowly in a course of 20–30 years or even longer, this brings the question of whether the brain can be rescued by the time of dementia, or whether treatments before the onset of dementia can prevent AD process [49]. Indeed, animal studies revealed that multiple transplantations of human umbilical cord-derived MSCs into 4-month-old SAMP8 mice (once a week for 8 weeks) greatly improved cognitive performance at 8 months, indicating that early treatment before AD onset is feasible [50, 51]. Moreover, a recent study also demonstrated that early inhibition of Ca^2+^ channels in pericytes could enhance brain energy supply and possibly cognitive function in AD mice [52]. Thus, in the present study, we treated *APOE*4/4 mice from 2–3 months old when the BBB damage begins to occur. We observed for the first time, that early and multiple transplantations of hiPSC-derived PCs could rescue AD phenotypes in aged *APOE*4/4 mice by preventing neuron loss, Aβ accumulation and p-tau formation, and improving BBB integrity. These data clearly support the notion that early intervention before the onset of dementia could effectively delay the progression of AD pathology.

In this study, we found rare intact *APOE*3/3-PCs in the brain after intravenous injection, but wide distribution of a huge number of ApoVs derived from *APOE*3/3-PCs in the brain as well as in the spleen, liver, kidneys and skin (data not shown). Our results also revealed that the in vivo distribution of ApoVs was not tissue- or organ-specific, although more ApoVs were observed in the brain than other tissues (data not shown). Interestingly, previous studies also demonstrated that ApoVs derived from MSCs are involved in the efficacy of MSC-based therapeutics [53, 54]. MSC-derived ApoVs have shown therapeutic effects in immune disorders, osteoporosis, skin injuries, hair regeneration and multiple myeloma [55–57]. However, whether ApoVs show therapeutic promise in AD treatment was unknown. Here, we found that the transplanted *APOE*3/3-PCs were primarily retained in the lungs of *APOE*4/4 mice and continuously released ApoVs. These ApoVs subsequently entered circulation, crossed the BBB and could be ingested by endogenous pericytes in *APOE*4/4 mouse brain. In vitro experiments also revealed that ApoVs derived from *APOE*3/3-PCs could efficiently prevent degeneration of *APOE*4/4 pericytes, and promote BBB function and Aβ transport. These results directly demonstrate the effectiveness of ApoVs^−PCs^ treatment. The functional recovery of endogenous pericytes and delayed progression of AD pathology in *APOE*4/4 mice may be primarily mediated by *APOE*3/3-PC-derived ApoVs.

Next, using transcriptomic and proteomic analysis, we identified IGF2 as a key effector molecule enriched in ApoVs^−PCs^, and found that IGF2 depletion substantially diminished the therapeutic effects of ApoVs^−PCs^ in vitro. IGF2 is a hormone that has a similar structure with insulin. It is highly expressed in mouse and human embryos, but the level declines dramatically after birth [58]. However, IGF2 is abundantly expressed in the CNS throughout life. IGF2 deficiency is associated with certain brain diseases, including AD, Parkinson’s disease, Huntington’s disease, and amyotrophic lateral sclerosis [59]. IGF2 can prevent dopaminergic neuronal loss and microglial over-activation [60, 61]. More recently, Pandey et al. reported that IGF2 is required for long-term memory and its level in hippocampal pericytes increases with learning [46]. The pericytes, choroid plexus and meninges are major sources of IGF2 in the brain. Selective knockout of IGF2 in pericytes leads to significant BBB permeability and impaired long-term memory [46, 62, 63]. Here, our results showed that the *APOE*3/3-PC-derived ApoVs contained high levels of IGF2 mRNA and protein and could circulate to the brain and be engulfed by neurons, pericytes and microglia. The IGF2 protein could provide immediate effects through direct signaling, while *IGF2* mRNA may support sustained action via ongoing translation. This “dual cargo” model enables ApoVs to exert both rapid and lasting effects. These results could partly explain why *APOE*3/3-PC transplantation prevented neuron loss and microglia activation. Notably, we found that IGF2 depletion in ApoVs largely abolished the therapeutic effects on *APOE*4/4-PCs in vitro, indicating IGF2 as a key regulator for pericyte homeostasis. Moreover, we also discovered that *APOE*3/3-PCs exhibited increased cell death, impaired BBB stability and Aβ clearance in vitro by IGF2 deletion. All these results above indicated that pericyte degeneration in *APOE*4/4 carriers may result from IGF2 decline. However, further studies are needed to uncover how IGF2 regulates pericyte homeostasis. In addition, previous studies demonstrated that IGF2 receptors are abundantly expressed on neurons, where they can directly bind recombinant IGF2 (r-IGF2), activate key neuroprotective pathways, thus preventing the loss of neurons and enhancing long-term memory formation [46, 60, 64]. Intracranial or stereotactic bilateral r-IGF2 administration significantly reduced the number of hippocampal Aβ plaques and improved memory in APP/PS1 mice [65, 66]. Interestingly, we found that neither r-IGF2 alone nor in combination with HDF-derived ApoVs could rescue *APOE*4/4-associated pericyte dysfunction in our co-culture system (data not shown). In contrast, IGF2 protein- and mRNA-enriched ApoVs derived from *APOE*3/3-pericytes could greatly improve *APOE*4/4-pericyte survival, barrier function and Aβ transport ability. These results underscore the importance of vesicle origin and cargo specificity in successful pericyte-targeted therapy. The discrepancy between our findings and previous reports may stem from differences in cell type (neuron vs pericyte) and experimental model (*APOE*4 vs APP/PS1).

## Conclusion

Taken together, we demonstrated that early and multiple transplantations of *APOE*3/3-PCs or *APOE*3/3-PC-derived IGF2-rich ApoVs could prevent pericyte degeneration and BBB damage, and rescue cognitive decline and AD pathologies in aged *APOE*4/4 mice, which may represent a promising therapy strategy for *APOE4*-related AD and other pericyte-degeneration disease.

## Supplementary Information

Additional file 1. **Table S1** Information for *APOE*3/3 or *APOE*4/4 carriers. **Figure S1**. Single *APOE*3/3-PCs injection did not rescue memory decline in aged APOE4/4 mice. **Figure S2**. *APOE*3/3-PCs transplantation rescued AD-related phenotypes in *APOE*4/4 mice. **Figure S3**. Assessments of microvascular length, perivascular macrophages and astrocytes after *APOE*3/3-PC transplantation. **Figure S4**. Distribution of pericytes in the lungs, liver, and spleen. **Figure S5**. *APOE*3/3-PCs generated huge number of ApoVs in *APOE*4/4 mice. **Figure S6**. Identification and pluripotency verification of hiPSCs in vitro. **Figure S7**. Comparison of the therapeutic effects of exosomes versus ApoVs on *APOE*4/4 PCs. **Figure S8**. IGF2 knockout promoted degeneration of *APOE*3/3 pericytes. **Figure S9**. Transplantation of ApoVs derived from *APOE*3/3-PCs rescued the cognitive decline in* APOE*4/4 mice, which was partially mediated by IGF2. **Figure S10**. Transplantation of ApoVs derived from *APOE*3/3-PCs alleviated the AD-related pathologies in *APOE*4/4 mice, which was partially mediated by IGF2. **Figure S11**. Transplantation of ApoVs derived from *APOE*3/3-PCs preserved BBB integrity in*APOE*4/4 mice, which was partially mediated by IGF2.

Additional file 2. **Table S2** Identified proteins in proteomics analysis.

Additional file 3. Uncropped Gels and Blots images.

## Data Availability

Data are available from the corresponding authors upon reasonable request.

## Abbreviations

AD: Alzheimer’s disease
BBB: Blood-brain barrier
hiPSCs: Human induced pluripotent stem cells
ApoVs: Apoptotic vesicles
IGF2: Insulin growth factor-2
Aβ: Amyloid-β
p-tau: Phosphorylated tau protein
HDFs: Human dermal fibroblasts
PCs: Pericyte-like cells
CNC: Cranial neural crest
tMCAO: Transient middle cerebral artery occlusion
PBMCs: Peripheral blood mononuclear cells
NCCM: Neural crest culture medium
OFT: Open field test
MWM: Morris water maze
NOR: New object recognition
IHC: Immunohistochemistry
IF: Immunofluorescence
EGF: Epidermal growth factor
bFGF: Basic fibroblast growth factor
CNS: Central nervous system
hESC: Human embryonic stem cell
MSC: Mesenchymal stem cell
EV: Extracellular vesicle
STS: Staurosporine
GO: Gene ontology
TEM: Transmission electron microscopy
HBMECs: Human brain microvascular endothelial cells
ADAD: Autosomal dominant AD
LOAD: Late-onset AD
CAA: Cerebral amyloid angiopathy
FITC: Fluorescein isothiocyanate
