# Supplementary material for: Transplantation of hiPSC-derived pericytes rescues Alzheimer’s disease phenotypes in APOE4/4 mice through IGF2-rich apoptotic vesicles
Source: Transl Neurodegener. 2025 Nov 13;14:57. doi: 10.1186/s40035-025-00512-6 (PMC12613509; doi:10.1186/s40035-025-00512-6)
Supplement: Supplementary file 1 — Additional file 1. Table S1 Information for APOE3/3 or APOE4/4 carriers. Figure S1. Single APOE3/3-PCs injection did not rescue memory decline in aged APOE4/4 mice. Figure S2. APOE3/3-PCs transplantation rescued AD-related phenotypes in APOE4/4 mice. Figure S3. Assessments of microvascular length, perivascular macrophages and astrocytes after APOE3/3-PC transplantation. Figure S4. Distribution of pericytes in the lungs, liver, and spleen. Figure S5. APOE3/3-PCs generated huge number of ApoVs in APOE4/4 mice. Figure S6. Identification and pluripotency verification of hiPSCs in vitro. Figure S7. Comparison of the therapeutic effects of exosomes versus ApoVs on APOE4/4 PCs. Figure S8. IGF2 knockout promoted degeneration of APOE3/3 pericytes. Figure S9. Transplantation of ApoVs derived from APOE3/3-PCs rescued the cognitive decline in APOE4/4 mice, which was partially mediated by IGF2. Figure S10. Transplantation of ApoVs derived from APOE3/3-PCs alleviated the AD-related pathologies in APOE4/4 mice, which was partially mediated by IGF2. Figure S11. Transplantation of ApoVs derived from APOE3/3-PCs preserved BBB integrity inAPOE4/4 mice, which was partially mediated by IGF2. [file 40035_2025_512_MOESM1_ESM.docx]

Supplementary Materials

**Transplantation of hiPSC-derived pericytes rescues** **Alzheimer’s disease phenotypes in APOE4/4 mice through** **IGF2-rich apoptotic vesicles**

Chuanfeng Xiong^1,2,3,#^, Yao Tang^2,3,#^, Junhua Chen^2,3^, Mingming Fan^2,3^, Lan Wei^2,3^, Zhaoran Dong^2,3^, Xingqiang Lai^2,3^, Xuejiao Men^5^, Qiumin Chen^2,3^, Dairui Li^2,3^, Wenjin Ye^2,3^, Yuanchen Ma^2,3^, Xiaoyong Chen^2,3^, Weijun Huang^2,3^, Zhengqi Lu^5^, Hong Chen^6^, Yunfeng Shen^1^, Yanming Chen^1,7,8*^, Andy Peng Xiang^2,3,4*^, Weiqiang Li^2,3,4*^

1. Department of Endocrinology and Metabolic Diseases, The Eighth Affiliated Hospital of Sun Yat-sen University, Shenzhen 518033, China.

2. Center for Stem Cell Biology and Tissue Engineering, Key Laboratory for Stem Cells and Tissue Engineering, Ministry of Education, Sun Yat-Sen University, Guangzhou 510080, China.

3. National-Local Joint Engineering Research Center for Stem Cells and Regenerative Medicine, Zhongshan School of Medicine, Sun Yat-sen University, Guangzhou 510080, China.

4. Department of Histoembryology and Cell Biology, Zhongshan School of Medicine, Sun Yat-sen University, Guangzhou 510080, China.

5. Department of Neurology, The Third Affiliated Hospital, Sun Yat-Sen University, Guangzhou 510630, China.

6. Center for Stem Cells Translational Medicine, Shenzhen Qianhai Shekou Free Trade Zone Hospital, Shenzhen 518067, China

7. Department of Endocrinology and Metabolic Diseases, The Third Affiliated Hospital of Sun Yat-sen University, Guangzhou 510630, China.

8. Guangdong Provincial Key Laboratory of Diabetology & Guangzhou Municipal Key Laboratory of Mechanistic and Translational Obesity Research, The Third Affiliated Hospital of Sun Yat-sen University, Guangzhou 510630, China.

^#^These authors contributed equally to this work.

***Corresponding authors:**

Weiqiang Li, Zhongshan School of Medicine, Sun Yat-sen University, No. 74, Zhongshan 2nd Road, Guangzhou, Guangdong Province 510080, China. E-mail: liweiq6@mail.sysu.edu.cn

Andy Peng Xiang, Zhongshan School of Medicine, Sun Yat-sen University, No. 74, Zhongshan 2nd Road, Guangzhou, Guangdong Province 510080, China. E-mail: [xiangp@mail.sysu.edu.cn](mailto:xiangp@mail.sysu.edu.cn)

Yanming Chen, Department of Endocrinology and Metabolic Diseases, The Eighth Affiliated Hospital of Sun Yat-sen University, Shenzhen, Guangdong, 518033, China. E-mail: [chyanm@mail.sysu.edu.cn](mailto:chyanm@mail.sysu.edu.cn)

**Table S1.** **Information for APOE3/3 or APOE4/4 carriers**

| Type | Sample source | Sex | Age | Clinical diagnosis |
| --- | --- | --- | --- | --- |
| APOE3/3 | PBMC | Female | 40 | no |
| APOE3/3 | PBMC | Female | 36 | no |
| APOE4/4 | PBMC | Female | 78 | AD |


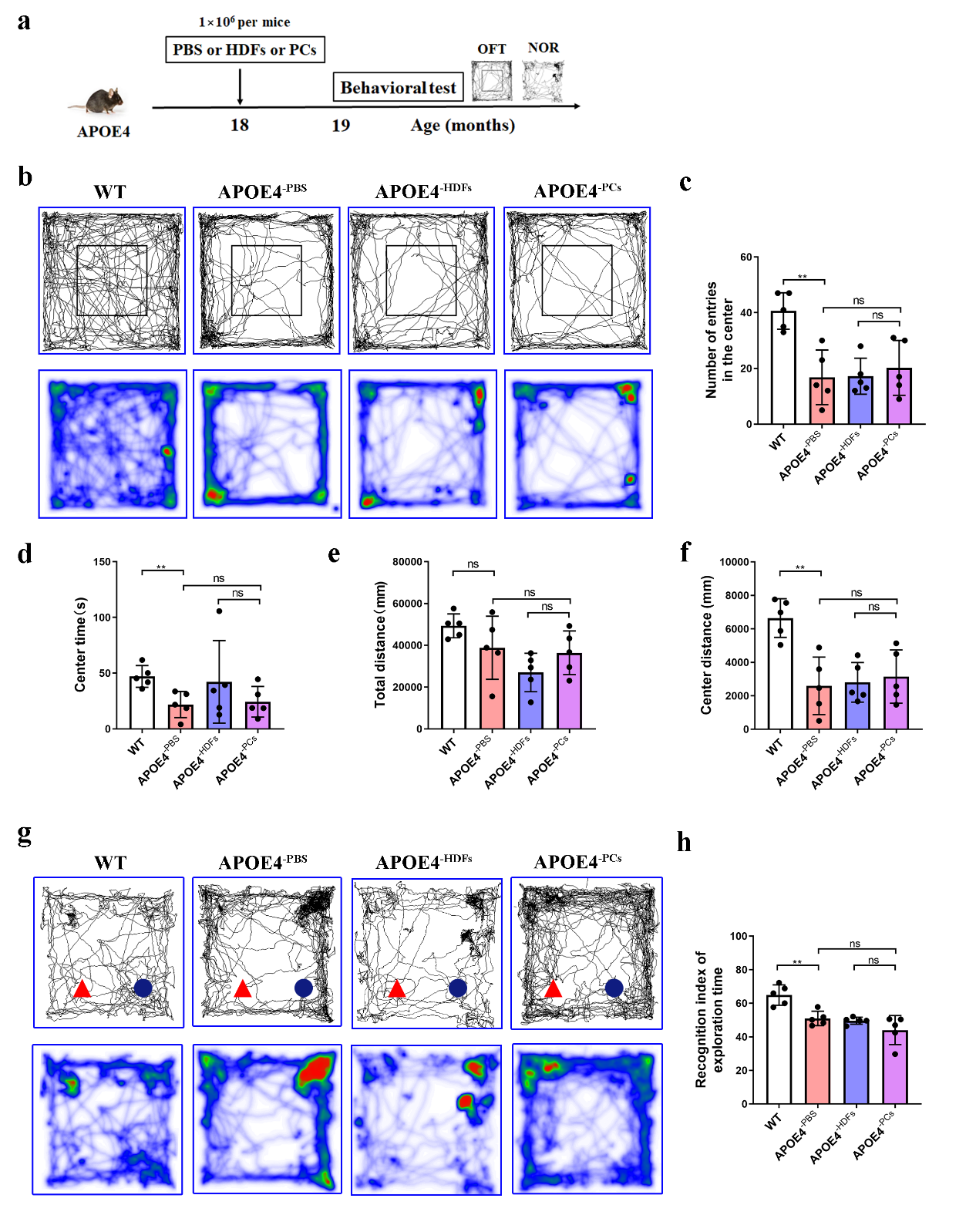


**Figure S1. Single** ***APOE*3/3-PCs injection did not rescue memory decline in aged *APOE*4/4 mice. a** Schematic illustrating the chronological order used for WT, PBS, HDFs or PCs treatment. Open filed test (OFT) and New object recognition (NOR) were used for behavioral test. **b-f** Representative traces and statistical analysis of OFT assay. (**b**) Tracing; (**c**) Number of entries in the center; (**d**) Time spent in center; (**e**) Total distance; (**f**) Center distance. **g-h** Representative traces and statistical analysis of NOR test. (**g**) Tracing; (**h**) Recognition index. All data are shown as means ± SD. *n*=5 mice per group. All comparisons were made by one-way ANOVA with Tukey's multiple comparison test; ns, non-significant, **P* < 0.05; ***P* < 0.01; ****P* < 0.001.


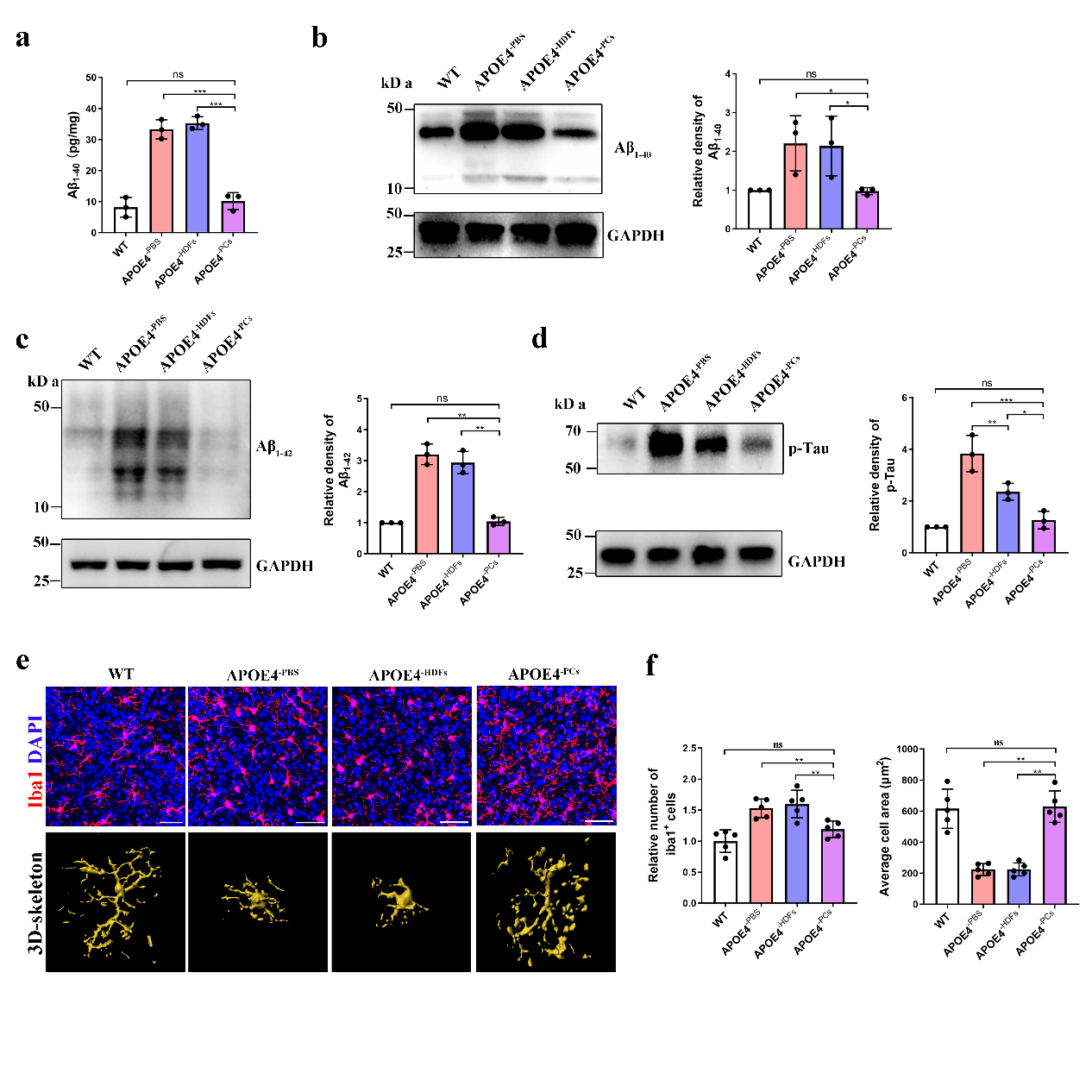


**Figure S2. *APOE*3/3-PCs transplantation rescued AD-related phenotypes in *APOE*4/4 mice. a** ELISA analysis for Aβ_1-40_ level in 18 months old mice brain between WT/PBS/HDFs/PCs groups (*n*=3 biological repeats for each group). **b-c** Images and statistical analysis of Aβ_1-40_ or Aβ_1-42_ deposition in different groups by western blot (*n*=3 biological repeats for each group). **d** Images and statistical analysis of p-Tau (T181) formation in different groups by western blot (*n*=3 biological repeats for each group). **e** Immunofluorescence staining of Iba1^+^ microglial cells between WT, PBS, HDFs and PCs group. Scale bar, 50μm. **f** Statistical analysis of relative number of Iba1^+^ cells and average cell area (μm^2^) (*n*=5 mice per group). All data are shown as means ± SD. All comparisons were made by one-way ANOVA with Tukey's multiple comparison test; ns, non-significant, **P* < 0.05; ***P* < 0.01; ****P* < 0.001.


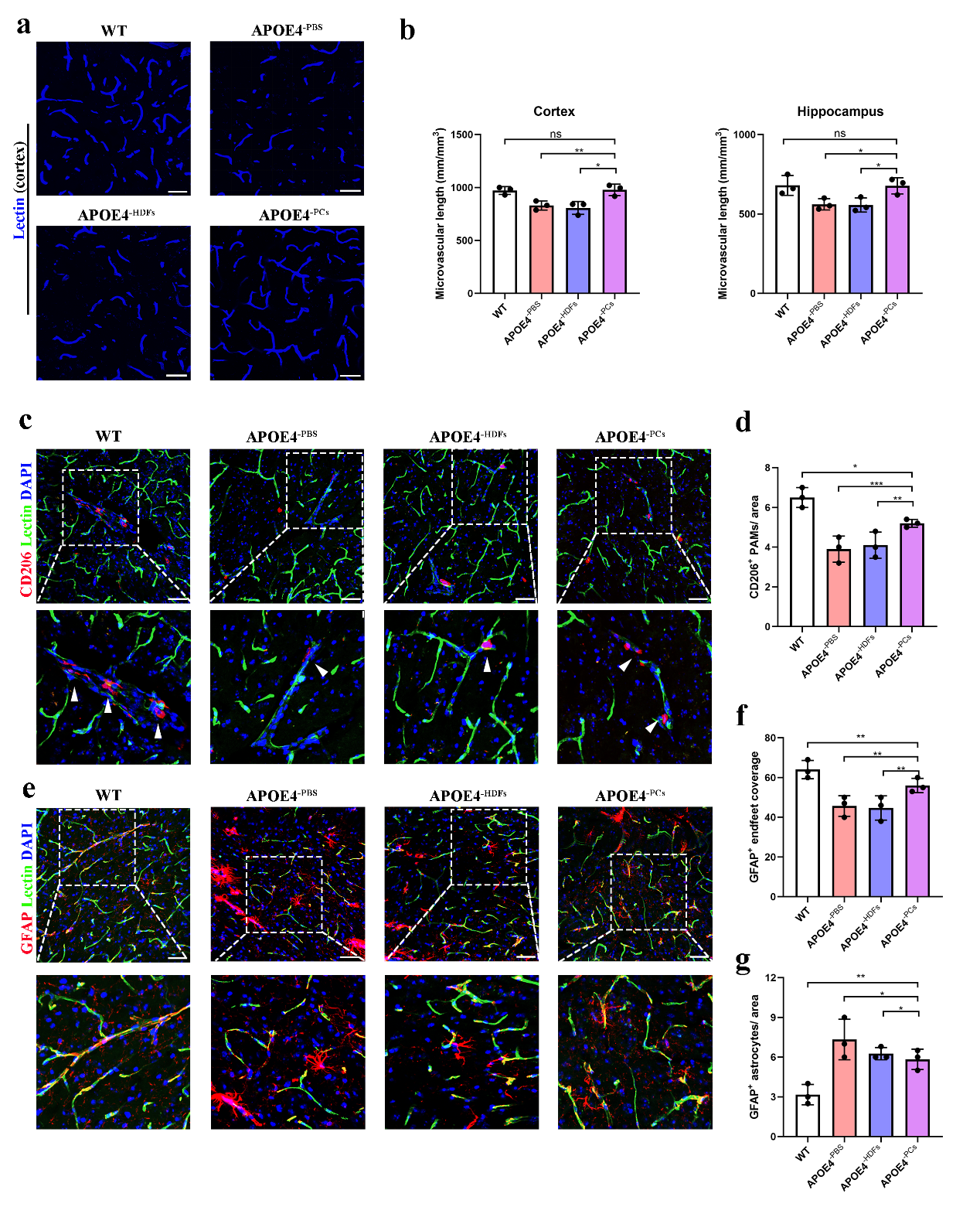


**Figure S3.** **Assessments of microvascular length, perivascular macrophages and astrocytes after *APOE*3/3-PC transplantation.** **a-b** Representative lectin^+^ endothelial profiles (blue) in the cortex and hippocampus. *n*=3 mice/group; scale bar, 50μm. **c-d** Representative CD206^+^ perivascular macrophages in the cortex. *n*=3 mice/group; scale bar, 50μm. **e-g** Representative GFAP^+^ astrocytes in the cortex. *n*=3 mice/group; scale bar, 50μm. All data are shown as means ± SD. All comparisons were made by one-way ANOVA with Tukey's multiple comparison test; ns, non-significant, **P* < 0.05; ***P* < 0.01; ****P* < 0.001.


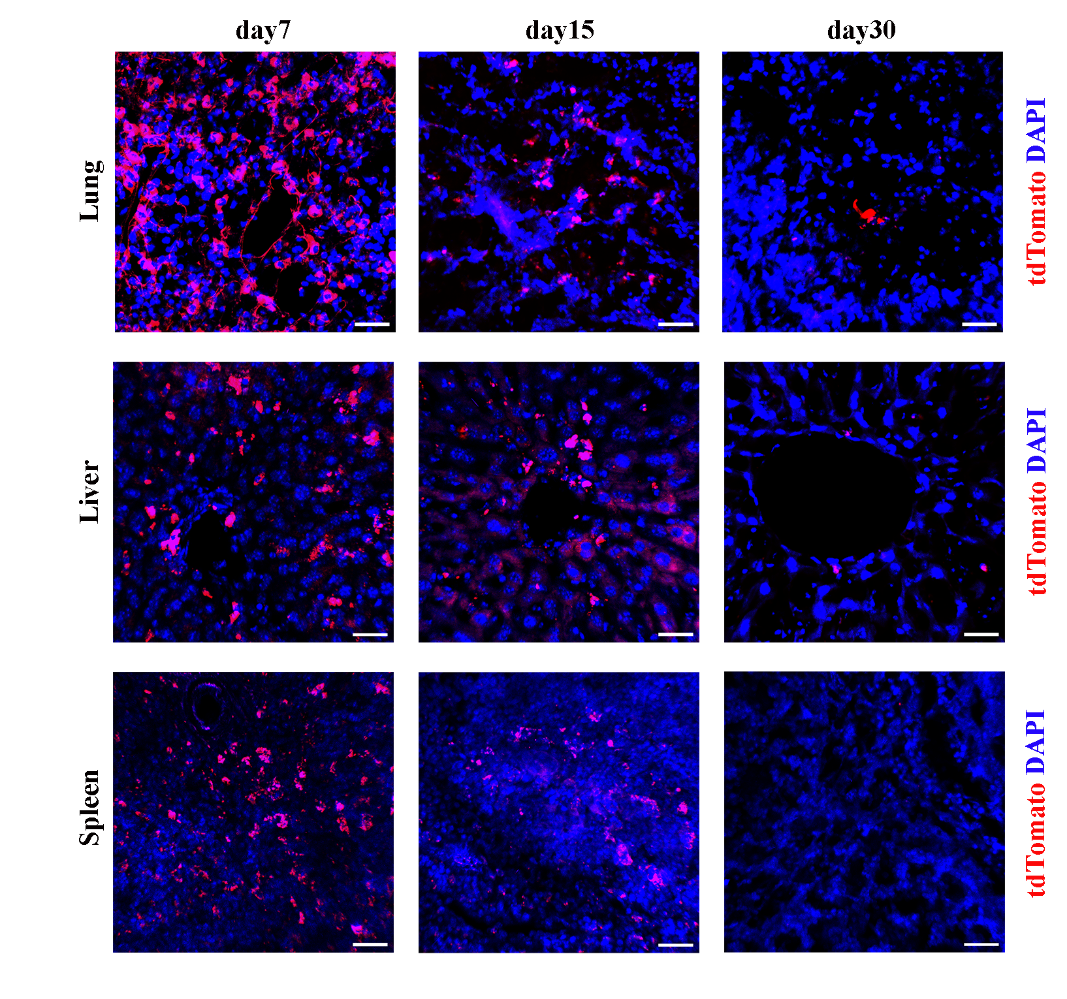


**Figure S4.** **Distribution of pericytes in the lungs, liver, and spleen.** Representative images of tdTomato^+^ pericyte distribution in APOE4/4 mice on days 7, 15, and 30 post-transplantations. Scale bar, 50μm.


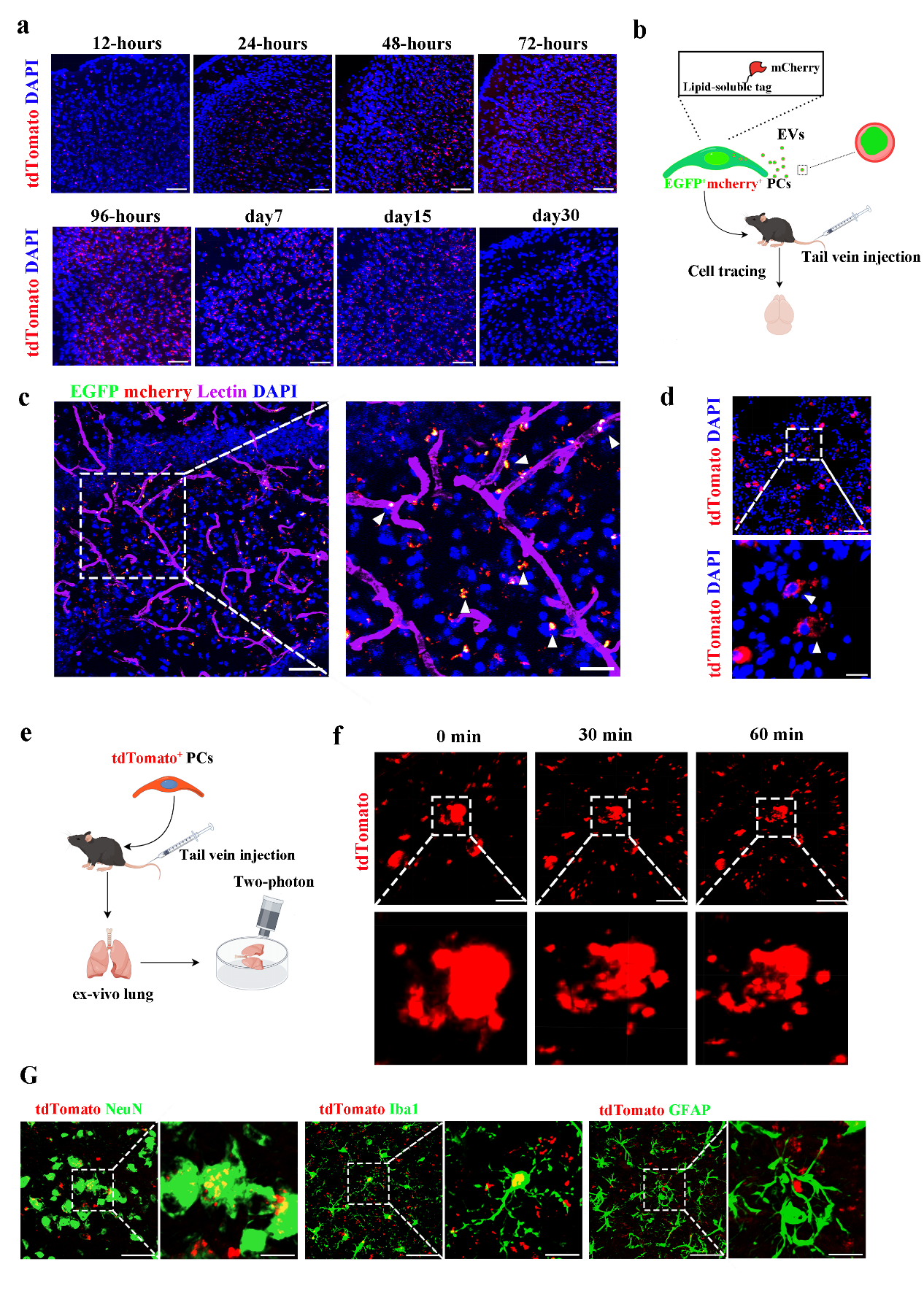


**Figure S5.** **APOE3/3-PCs generated huge number of ApoVs in APOE4/4 mice. a** Images represent of tdTomato^+^ signals in brain of 9-month-old APOE4/4 mice at different timepoints. Scale bar, 50μm. **b** Strategies to generate mCherry^+^EGFP^+^ PCs for cell tracing. **c** Representative images of EGFP^+^ signal covered by mCherry^+^ signal. Scale bar (left), 50μm. Scale bar (right), 20μm. **d** tdTomato^+^ PCs tracing in lung of 9-month-old *APOE4/4* mice. Scale bar (up), 50μm. Scale bar (down), 20μm. **e** Schematic diagram for detection of ApoVs releasing from *ex-vivo* cultured lung tissue 3 days post-transplantation using multiphoton microscope. **f** Representative images for ApoVs releasing from lung in a time series. Scale bar, 15μm. **g** Representative images of ApoVs engulfed by NeuN^+^ neurons, Iba1^+^ microglials and GFAP^+^ astrocytes. Scale bar (left), 50μm. Scale bar (right), 20μm. *n*=3 mice per group.


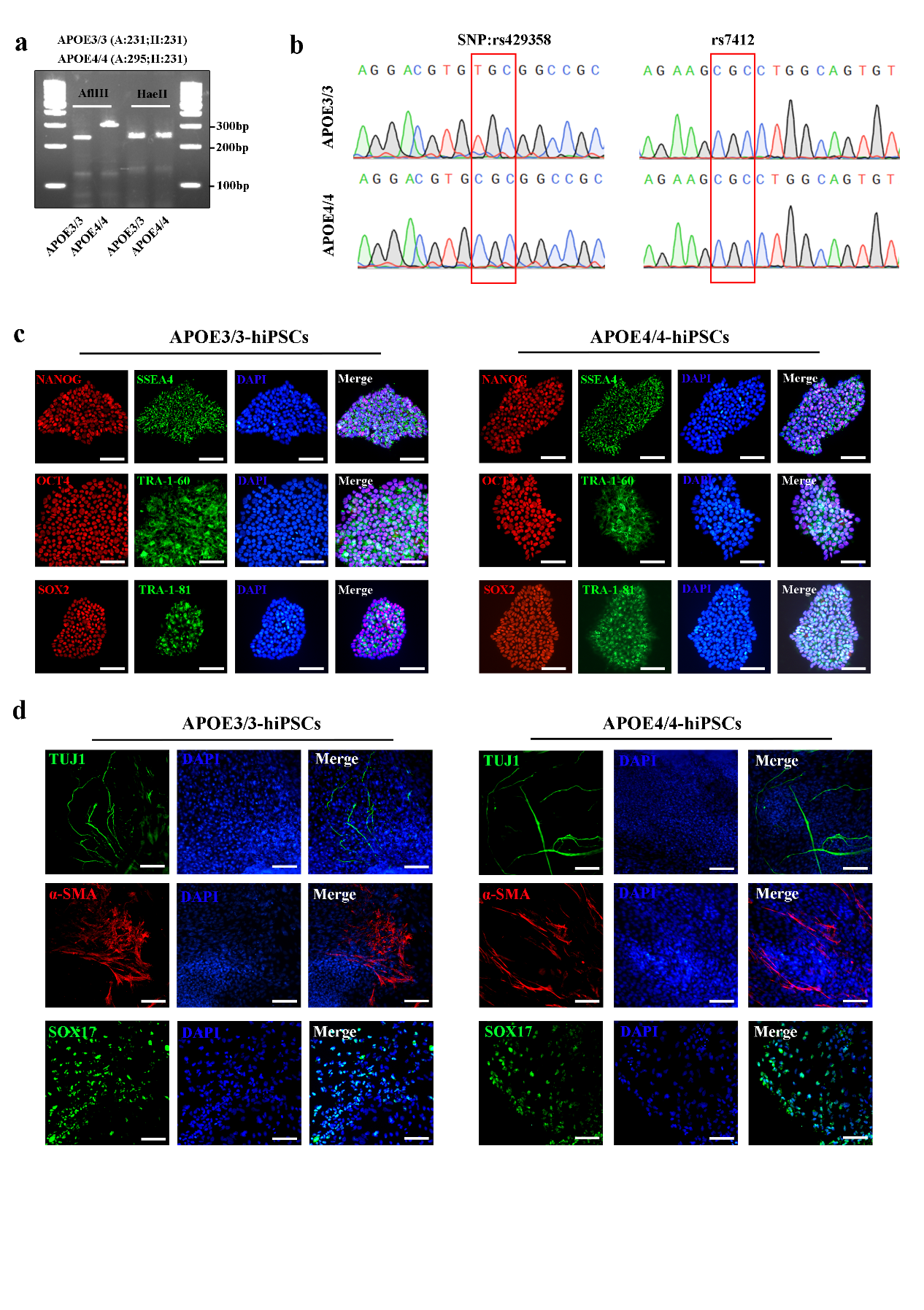


**Figure S6. Identification and pluripotency verification of hiPSCs *in vitro*. a** *APOE* genotyping was performed using PCR-restriction fragment length polymorphism approach. PCR products were digested by either 2.5 UAfIll or 1.5 U Haell at 37°C overnight. The digested products were analyzed on a 4% agarose gel. **b** Sanger sequencing of PCR products. **c** *APOE3/3* or *APOE4/4* hiPSCs expressed pluripotency markers including OCT4, SOX2, NANOG, SSEA4, TRA-1–60, and TRA-1–81. Scale bar, 50μm. **d** *In vitro* differentiation of *APOE3/3* or *APOE4/4* hiPSCs using a trilineage differentiation kit. Scale bar, 50μm.


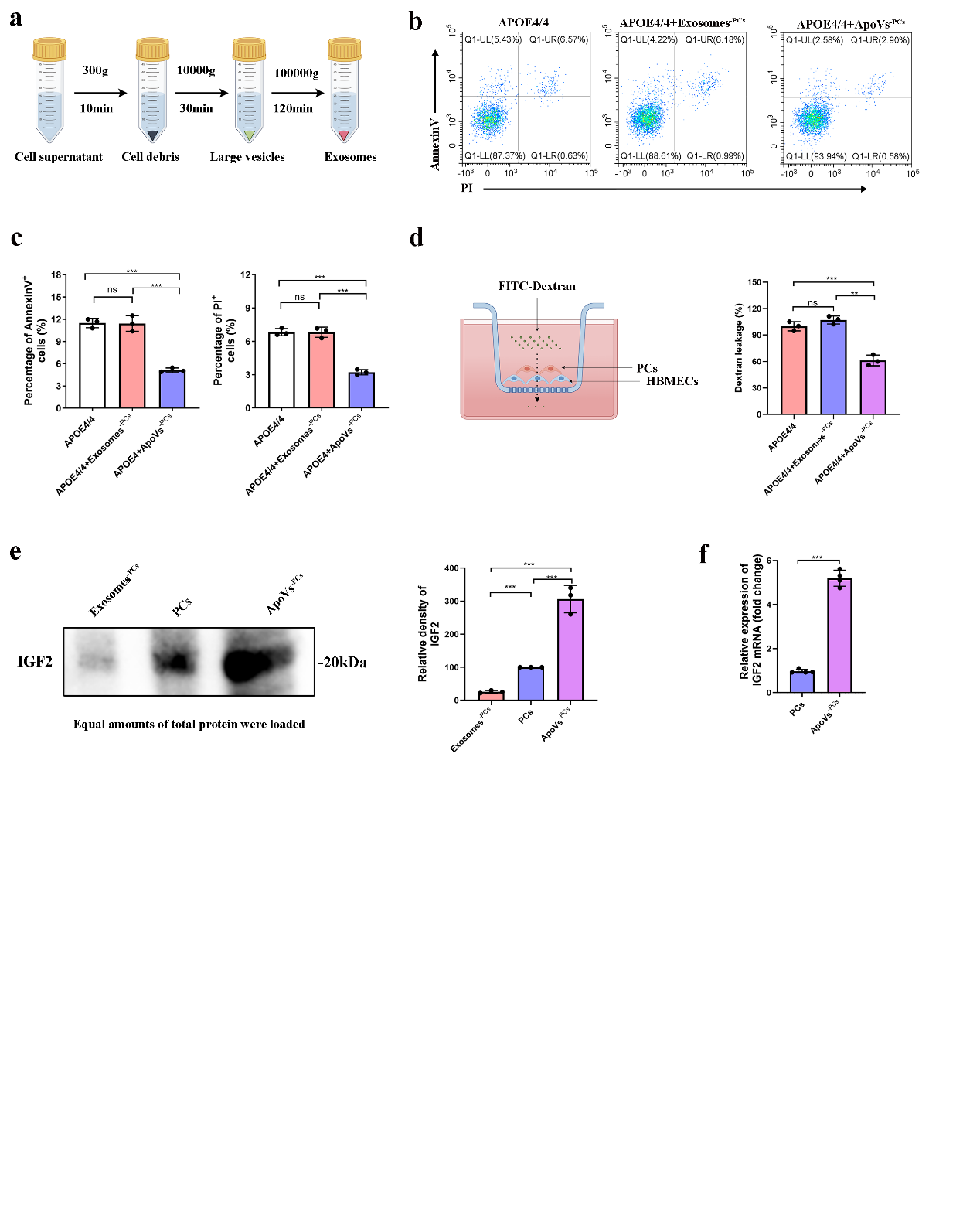


**Figure S7*.*** **Comparison of the therapeutic effects of exosomes versus ApoVs on *APOE*4/4 PCs. a** Strategies to harvest exosomes. **b-c** Flow cytometry analysis and quantification of *APOE4/4*-PCs apoptosis rate using AnnexinV/PI staining after exosomes or ApoVs treatment for 48 hours (*n*=3 biological repeats for each group). **d** Dextran leakage assay was performed after exosomes or ApoVs treatment (*n*=3 biological repeats for each group). **e** Western blot was used to detected IGF2 protein levels between exosomes, *APOE3/3*-pericytes and ApoVs with equal loaded total protein amounts (*n*=3 biological repeats for each group). **f** qPCR was used to detected *IGF2* mRNA levels between *APOE3/3*-pericytes and ApoVs (*n*=3 biological repeats for each group). All comparisons were made by one-way ANOVA with Tukey’s multiple comparison test or Student’s t tests; ns, non-significant, **P* < 0.05; ***P* < 0.01; ****P* < 0.001).


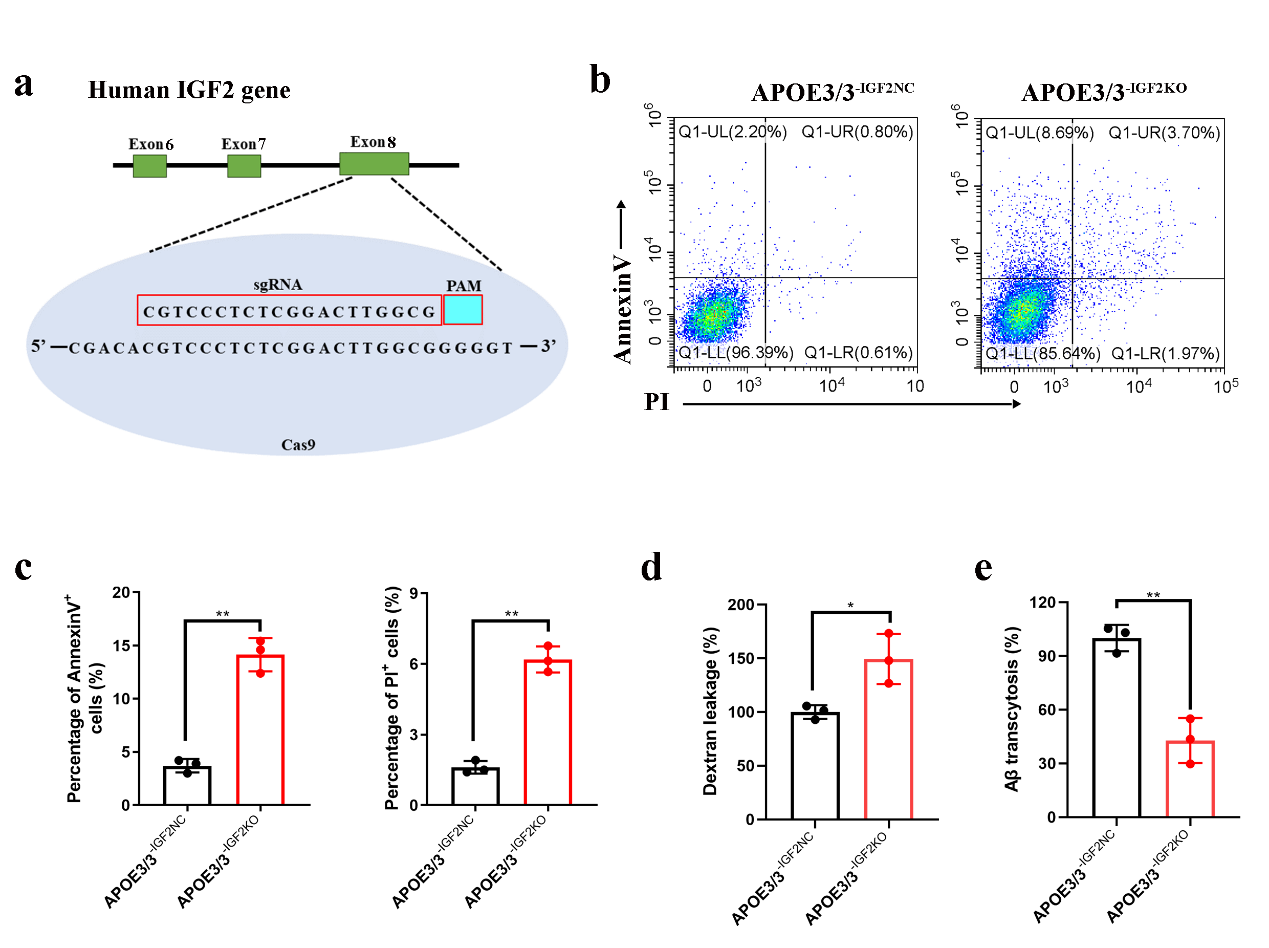


**Figure S8.** **IGF2 knockout promoted degeneration of APOE3/3 pericytes***.* **a** Design strategy of IGF2 sgRNA. **b** AnnexinV/PI staining showed the cell death rate in *APOE3/3*^-IGF2NC^ and *APOE3/3*^-IGF2KO^. **c** Statistical analysis for the number of Annexin V^+^ or PI ^+^ cells. **d** Dextran leakage analysis in *APOE3/3*^-IGF2NC^ and *APOE3/3*^-IGF2KO^ groups. **e** Aβ transcytosis analysis in *APOE3/3*^-IGF2NC^ and *APOE3/3*-^IGF2KO^ groups (*n*=3 biological repeats for each group). Student’s t tests were used for comparisons of the mean between two groups; **P* < 0.05; ***P* < 0.01.


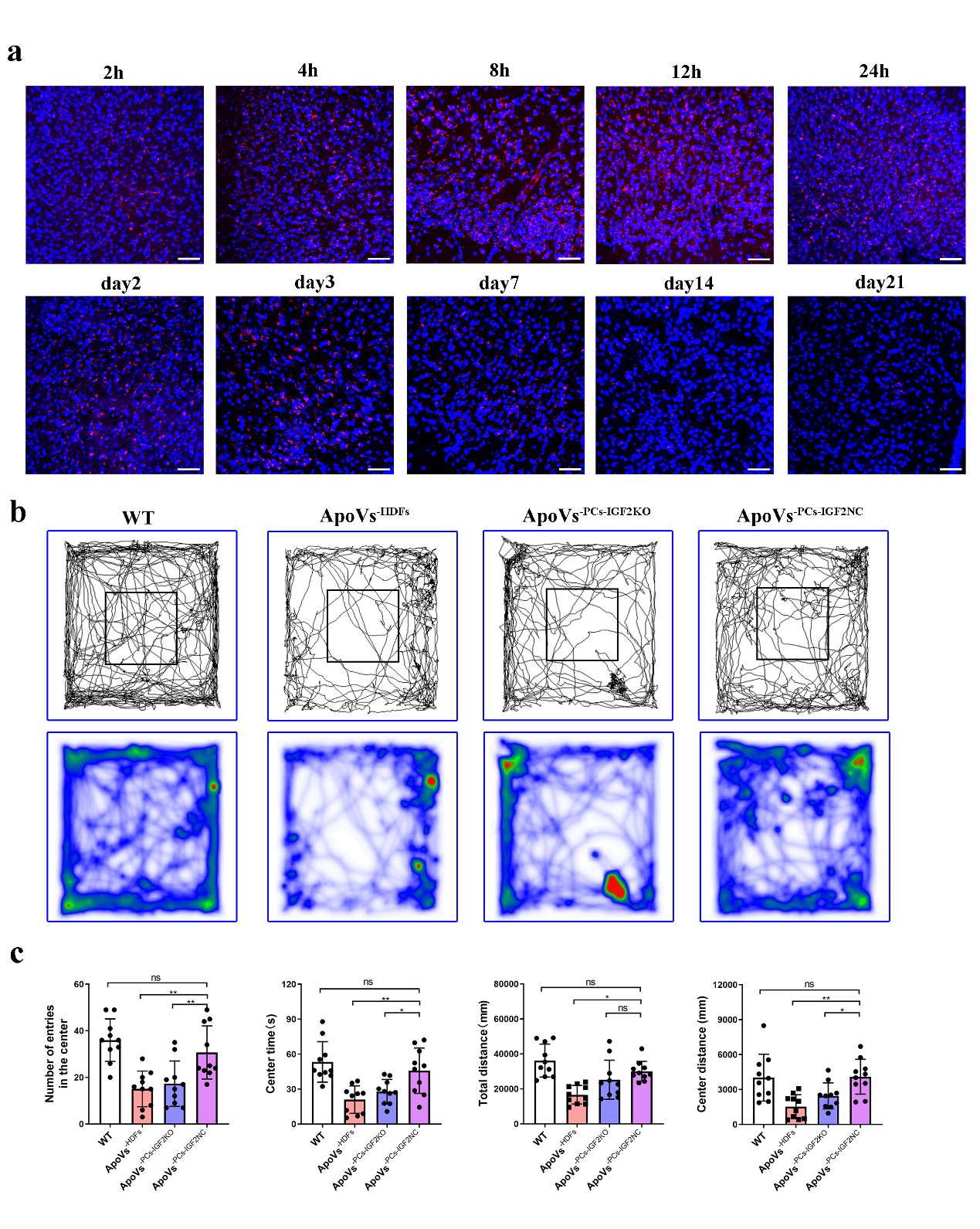


**Figure S9.** **Transplantation of ApoVs derived from *APOE*3/3-PCs rescued the cognitive decline in *APOE*4/4 mice, which was partially mediated by IGF2. a** Tracing of tdTomato^+^ ApoVs in 9-months old *APOE4/4* mice brain at different timepoints Scale bar, 50μm. **b-c** Representative traces and statistical analysis of OFT assay. (**b**) Tracing. (**c**) Number of entries in the center, Time spent in center, Total distance and Center distance. *n*=9-10 mice per group for behavioral test. All comparisons were made by one-way ANOVA with Tukey's multiple comparison test; ns, non-significant, **P* < 0.05; ***P* < 0.01; ****P* < 0.001.


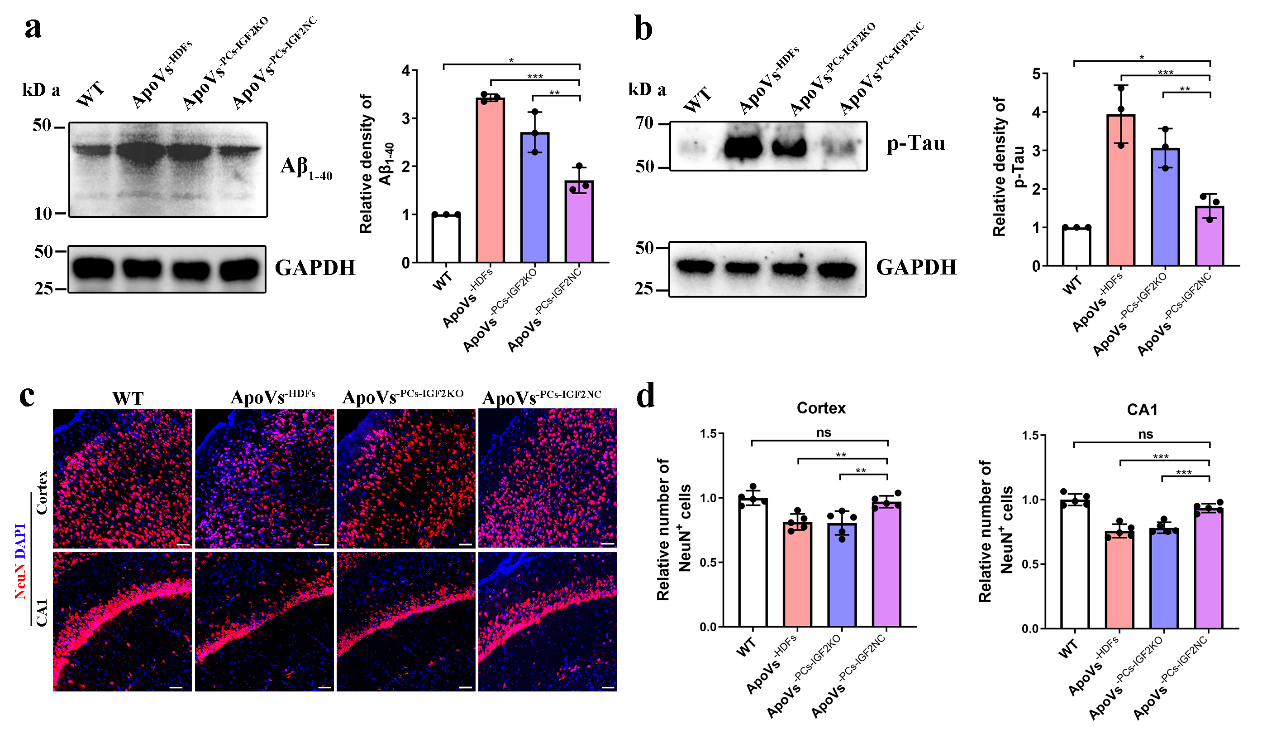


**Figure S10.** **Transplantation of ApoVs derived from APOE3/3-PCs alleviated the AD-related pathologies in APOE4/4 mice, which was partially mediated by IGF2. a** Images and statistical analysis of Aβ_1-40_ deposition by western blot (n=3 biological repeats for each group). **b** Images and statistical analysis of p-Tau (T181) formation by western blot (*n*=3 biological repeats for each group). **c** Detection of NeuN^+^ cells in different groups by immunofluorescence staining. Scale bar, 50μm. **d** Quantification analysis of NeuN^+^ neuron cells in different groups in the specific region of Cortex and CA1 area. *n*=5 mice per group. All comparisons were made by one-way ANOVA with Tukey's multiple comparison test; ns, non-significant, **P* < 0.05; ***P* < 0.01; ****P* < 0.001.


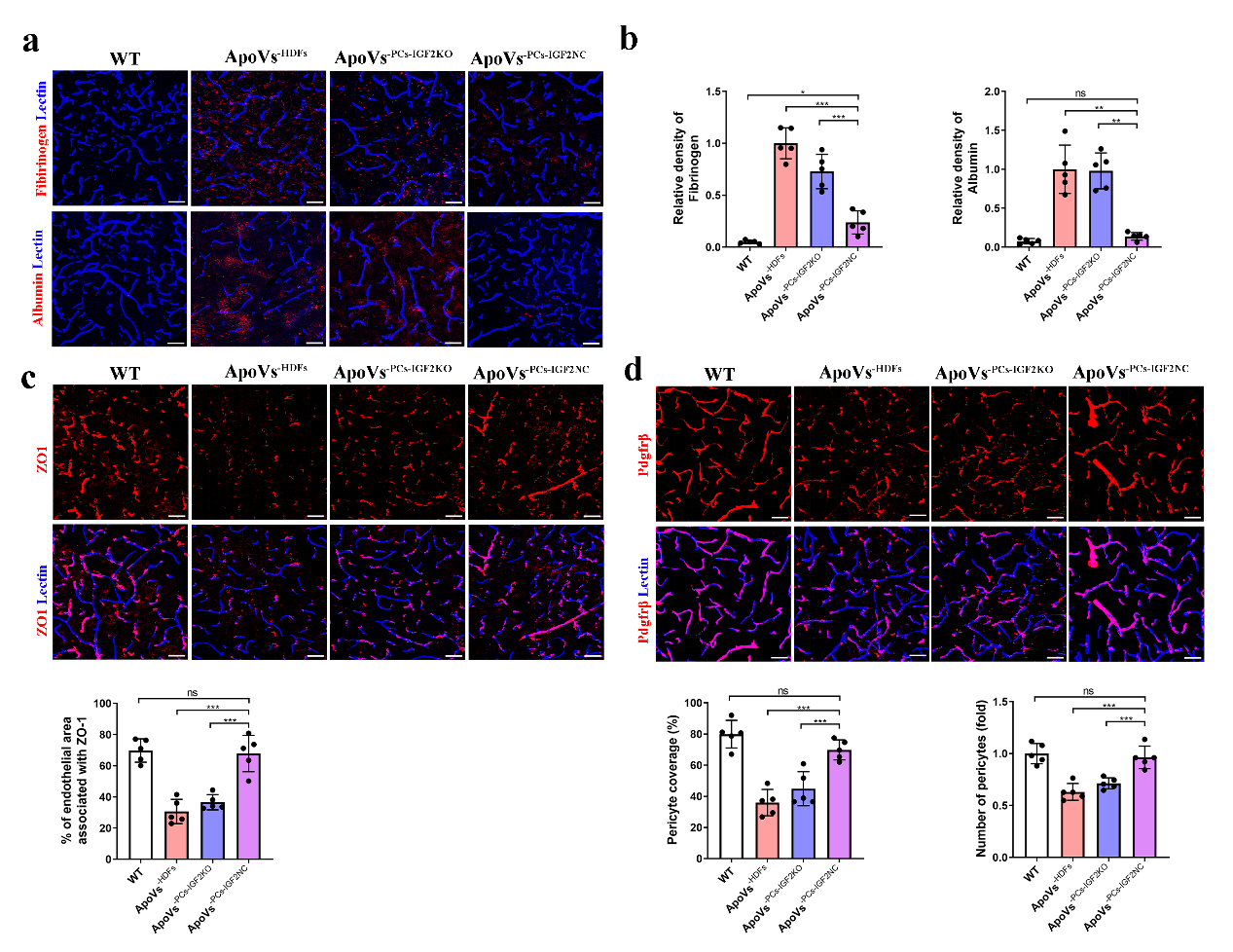


**Figure S11.** **Transplantation of ApoVs derived from *APOE*3/3-PCs preserved BBB integrity in *APOE*4/4 mice, which was partially mediated by IGF2.** **a-b** Confocal microscopy and quantification analysis of the extravascular fibrinogen (red) and albumin (red) leakage through lectin-labeled (bule) capillaries. Scale bar, 50μm. **c** Confocal microscopy and quantification analysis of the lectin-labeled (bule) capillaries covered by tight junction protein ZO1 (red). Scale bar, 50μm. **d** Detection of pdgfrβ^+^ pericytes in mouse brain. Scale bar, 50μm. The percentage of Lectin^+^ capillaries covered by pdgfrβ^+^ pericytes and relative number of pdgfrβ^+^ pericytes were quantified. All data are shown as means ± SD. *n*=5 mice per group. All comparisons were made by one-way ANOVA with Tukey's multiple comparison test; ns, non-significant, **P* < 0.05; ***P* < 0.01; ****P* < 0.001.
