# Supplementary figures and images for: Transplantation of hiPSC-derived pericytes rescues Alzheimer’s disease phenotypes in APOE4/4 mice through IGF2-rich apoptotic vesicles
Source: Transl Neurodegener. 2025 Nov 13;14:57. doi: 10.1186/s40035-025-00512-6 (PMC12613509; doi:10.1186/s40035-025-00512-6)

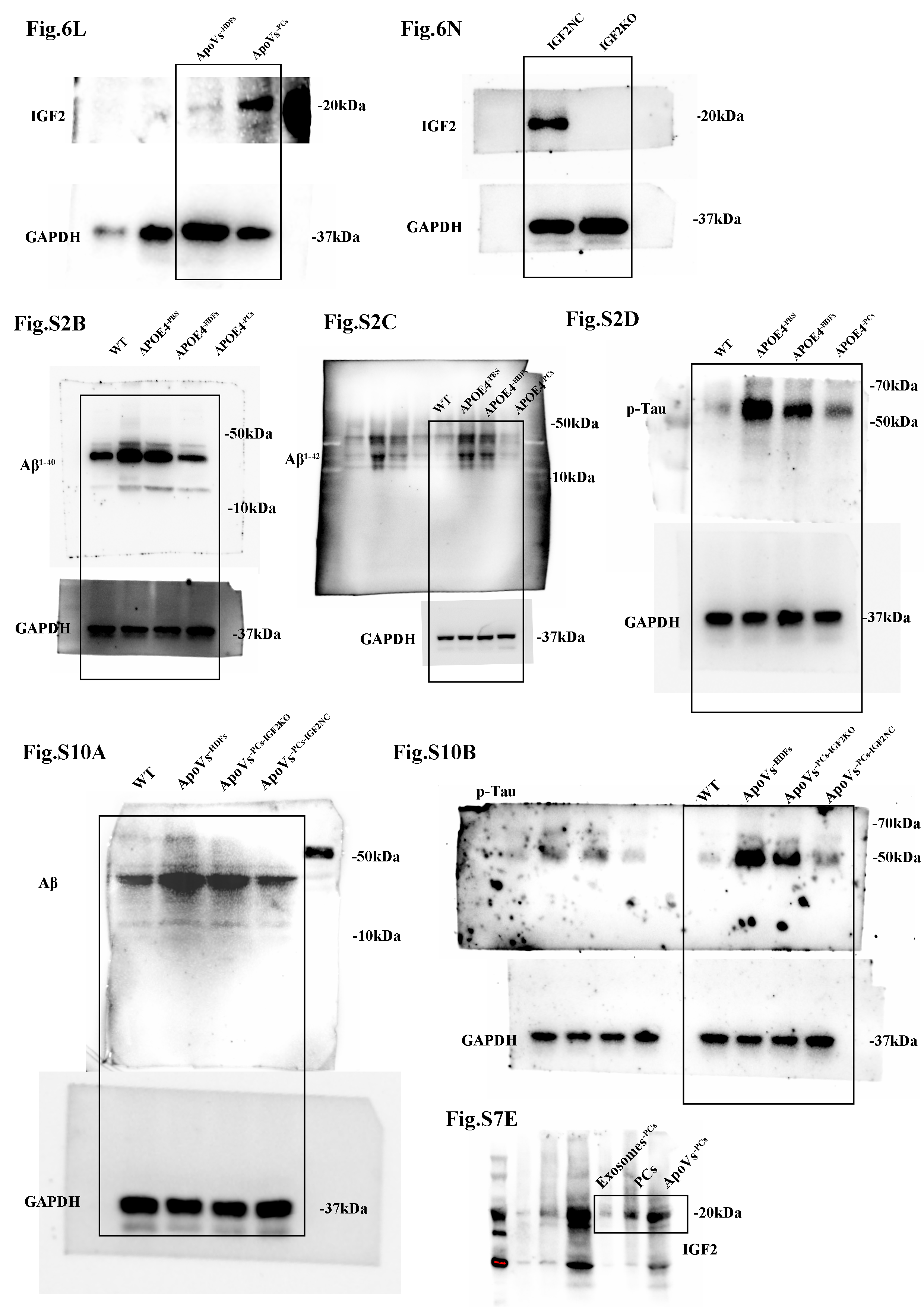

Supplement: Supplementary file 3 — Additional file 3. Uncropped Gels and Blots images. [file 40035_2025_512_MOESM3_ESM.png]
